# Supplementary material for: An HIF-1α/VEGF-A Axis in Cytotoxic T Cells Regulates Tumor Progression
Source: Cancer Cell. 2017 Nov 13;32(5):669–683.e5. doi: 10.1016/j.ccell.2017.10.003 (PMC5691891; doi:10.1016/j.ccell.2017.10.003)
Supplement: Document S1. Figures S1–S6 and Tables S1–S3 [file mmc1.pdf]

Cancer Cell, Volume *32*

Supplemental Information

An HIF-1α/VEGF-A Axis in Cytotoxic T Cells Regulates Tumor Progression

Asis Palazon, Petros A. Tyrakis, David Macias, Pedro Veliça, Helene Rundqvist, Susan Fitzpatrick, Nikola Vojnovic, Anthony T. Phan, Niklas Loman, Ingrid Hedenfalk, Thomas Hatschek, John Lövrot, Theodoros Foukakis, Ananda W. Goldrath, Jonas Bergh, and Randall S. Johnson

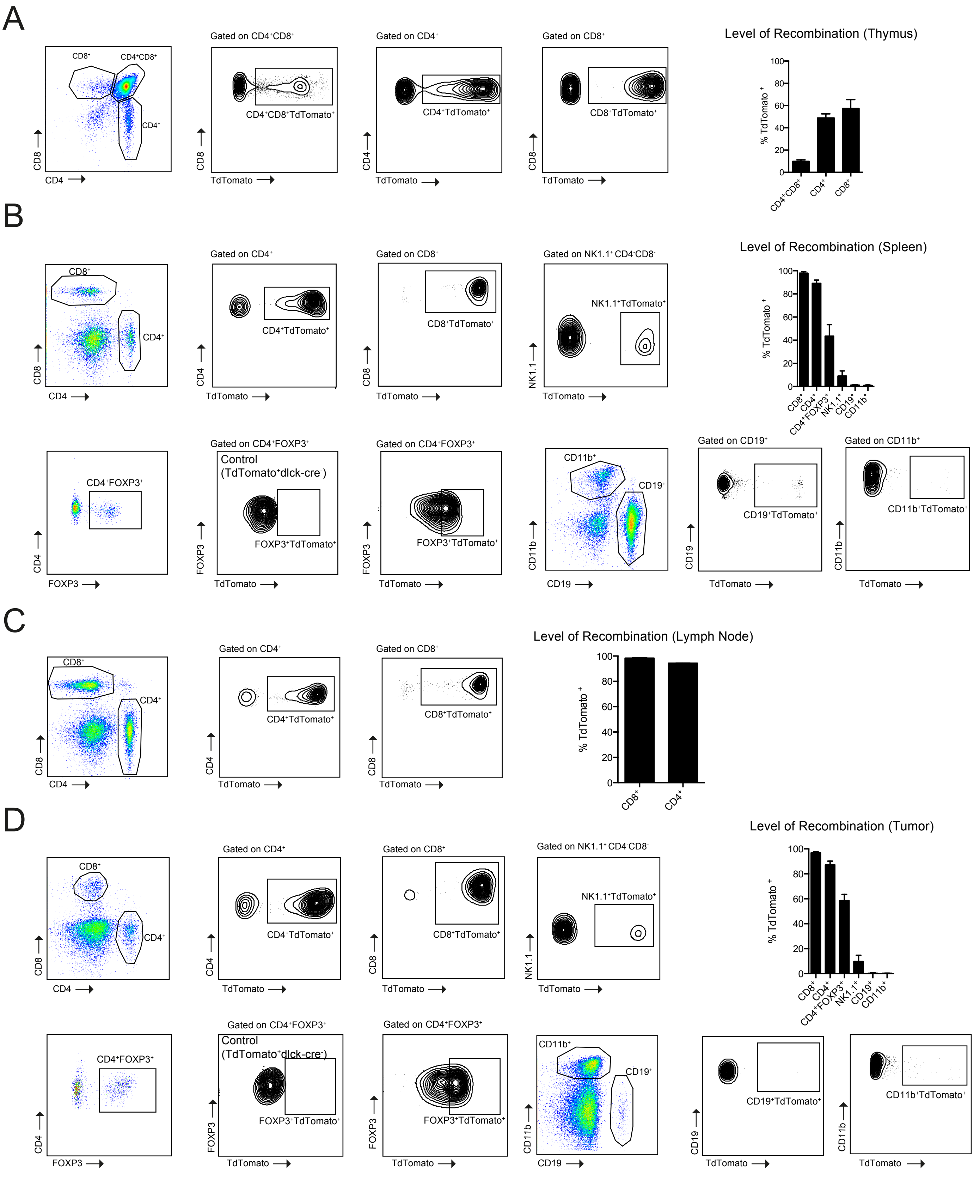

Figure S1, related to Figure 1.
(A-D) Level of dlck-cre recombination in thymus (A), spleen (B), lymph nodes (C), and subcutaneous LLC tumors (D) (n=4) was measured using a TdTomato reporter mouse line by flow cytometry. Error bars represent SD.

Table S1, related to Figure 1.

	Mean pO_2 (mmHg)	SD
Culture media 21% O_2	107.38	2.65
Culture media 5% O_2	31.65	0.26
Culture media 1% O_2	10.33	4.25
Spleen	21.73	12.6
Tumor (LLC)	1.95	2.41

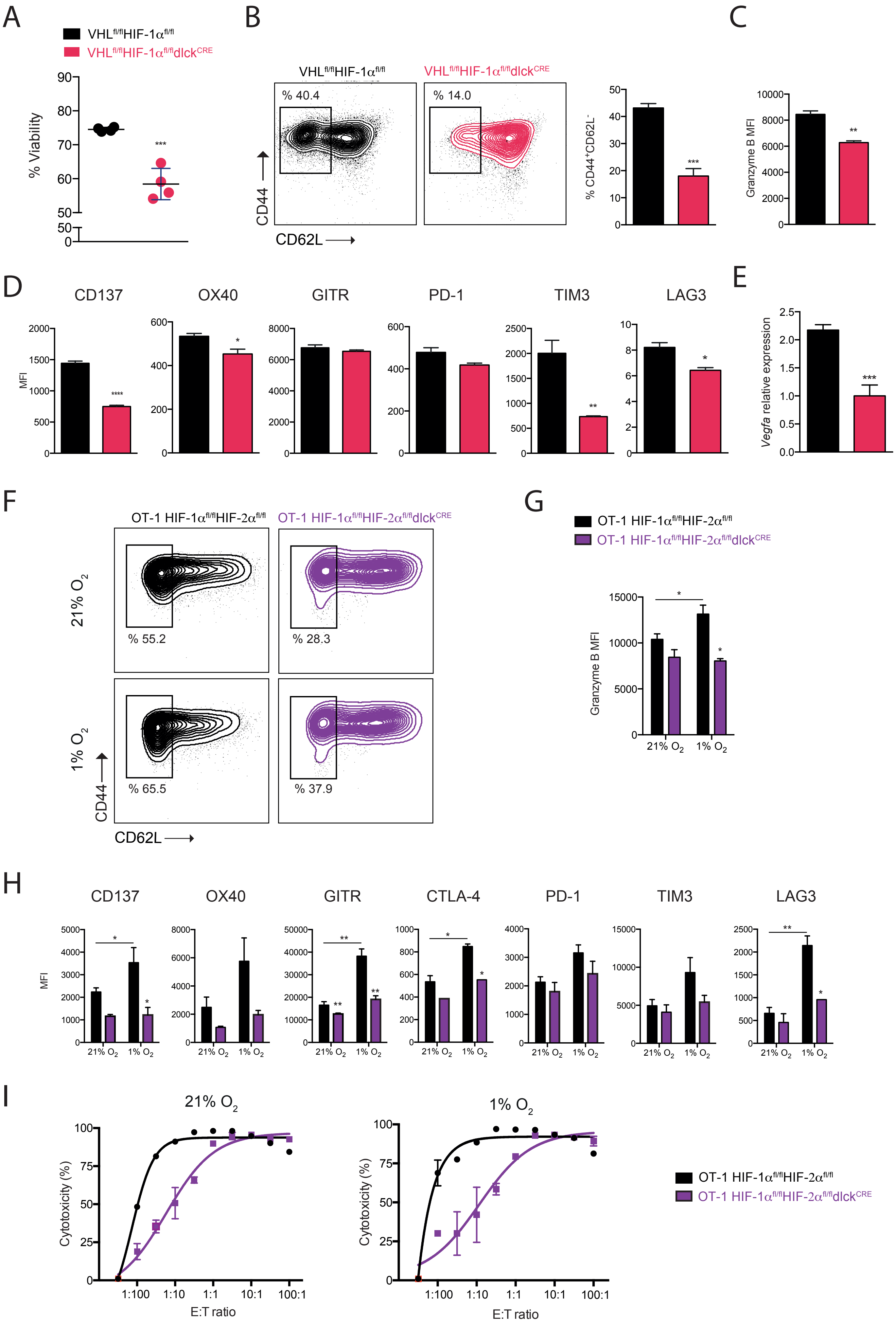

A
VHL$^{fl/fl}$HIF-1α$^{fl/fl}$
VHL$^{fl/fl}$HIF-1α$^{fl/fl}$dlck^{CRE}
% Viability
***
B
VHL$^{fl/fl}$HIF-1α$^{fl/fl}$
VHL$^{fl/fl}$HIF-1α$^{fl/fl}$dlck^{CRE}
% 40.4
% 14.0
CD44
CD62L
% CD44^{+}CD62L^{-}
***
C
Granzyme B MFI
**
D
CD137
OX40
GITR
PD-1
TIM3
LAG3
MFI
****
*
**
*
E
Vegfa relative expression
***
F
OT-1 HIF-1α$^{fl/fl}$HIF-2α$^{fl/fl}$
OT-1 HIF-1α$^{fl/fl}$HIF-2α$^{fl/fl}$dlck^{CRE}
21% O_2
1% O_2
% 55.2
% 28.3
% 65.5
% 37.9
CD44
CD62L
G
OT-1 HIF-1α$^{fl/fl}$HIF-2α$^{fl/fl}$
OT-1 HIF-1α$^{fl/fl}$HIF-2α$^{fl/fl}$dlck^{CRE}
Granzyme B MFI
21% O_2
1% O_2
H
CD137
OX40
GITR
CTLA-4
PD-1
TIM3
LAG3
MFI
21% O_2
1% O_2
I
21% O_2
1% O_2
Cytotoxicity (%)
E:T ratio
1:100
1:10
1:1
10:1
100:1
OT-1 HIF-1α$^{fl/fl}$HIF-2α$^{fl/fl}$
OT-1 HIF-1α$^{fl/fl}$HIF-2α$^{fl/fl}$dlck^{CRE}

Figure S2, related to Figure 2.

(A-E) $CD8^+$ T cells were isolated from spleens of $VHL^{fl/fl}HIF\text{-}1\alpha^{fl/fl}dlck^{CRE}$ (pink) and littermate control (black) mice, activated with αCD3/αCD28 for 48 hr, and then expanded for 5 days in the presence of IL-2: percent survival (A), expression of CD44 and CD62L by flow cytometry (B), expression of Granzyme B by intracellular flow cytometry (C), surface expression of costimulatory molecules/checkpoint receptors CD137, OX40, GITR, PD-1, TIM-3 and LAG3 by flow cytometry (D) and relative expression of *Vegfa* by QRT-PCR (E), n=4, error bars represent SD. (F-H) $CD8^+$ T cells were isolated from spleens of OT-1 $HIF\text{-}1\alpha^{fl/fl}HIF\text{-}2\alpha^{fl/fl}dlck^{CRE}$ (purple) and littermate control (black) mice, activated with cognate peptide for 2 days, and then expanded for 5 days in the presence of IL-2 and subjected to 21% or 1% O_2 for 24 hr: expression of CD44 and CD62L by flow cytometry (F) expression of Granzyme B by intracellular flow cytometry (G), expression of the indicated costimulatory molecules/checkpoint receptors by flow cytometry (H), n=4, error bars represent SD. (I) *In vitro* cytotoxicity assay: EG7-OVA target cells and control or mutant OT-1 $CD8^+$ T cells were co-cultured at different effector to target (E:T) ratios for 24 hr under 21% or 1% O_2 (n=2, error bars represent SE).

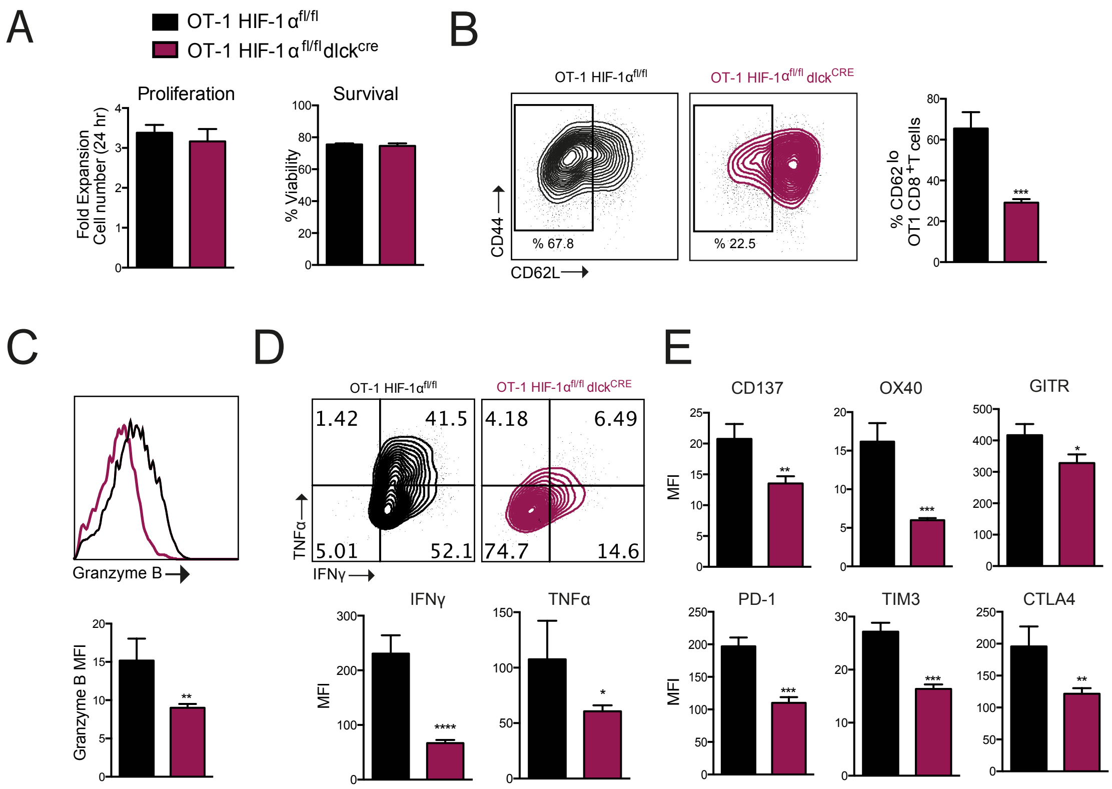

A
OT-1 HIF-1α fl/fl
OT-1 HIF-1α fl/fl dlckcre
Proliferation
Fold Expansion Cell number (24 hr)
Survival
% Viability
B
OT-1 HIF-1αfl/fl
OT-1 HIF-1αfl/fl dlckCRE
% 67.8
% 22.5
CD44
CD62L
% CD62 lo OT1 CD8+T cells
C
Granzyme B
Granzyme B MFI
D
1.42
41.5
5.01
52.1
4.18
6.49
74.7
14.6
TNFα
IFNγ
MFI
E
CD137
OX40
GITR
PD-1
TIM3
CTLA4
MFI

Figure S3, related to Figure 4.

(A-B) Splenic OT-1 cells were activated *in vitro* with cognate SIINFEKL peptide and expanded under 21% O_2 for 3 days in the presence of IL-2: proliferation and survival (A), surface CD62L expression by flow cytometry (B), (n=3, error bars represent SD). (C) Intracellular granzyme B expression (n=3, error bars represent SD). (D) Intracellular TNFα and IFNγ expression after 4 hr *in vitro* restimulation with SIINFEKL (n=3, error bars represent SD). (E) Surface expression of costimulatory/checkpoint receptors CD137, OX40, GITR, PD-1 and TIM3 and intracellular expression of CTLA4 (n=3, error bars represent SD). Two-tailed student T test was used for comparisons.

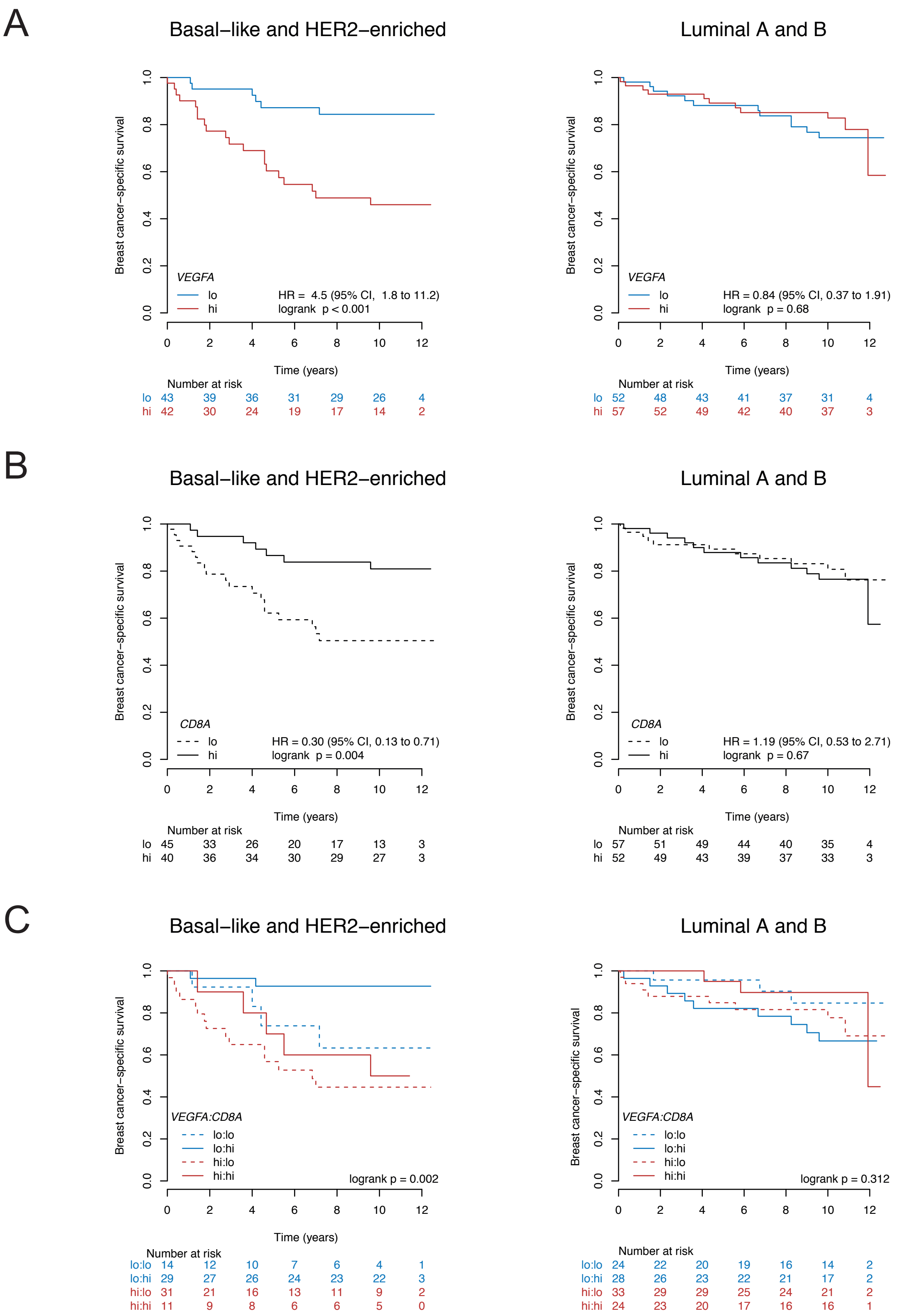

A
Basal–like and HER2–enriched
Luminal A and B
Breast cancer–specific survival
VEGFA
lo
hi
HR = 4.5 (95% CI, 1.8 to 11.2)
logrank p < 0.001
HR = 0.84 (95% CI, 0.37 to 1.91)
logrank p = 0.68
Time (years)
Number at risk
lo 43 39 36 31 29 26 4
hi 42 30 24 19 17 14 2
lo 52 48 43 41 37 31 4
hi 57 52 49 42 40 37 3
B
Basal–like and HER2–enriched
Luminal A and B
CD8A
HR = 0.30 (95% CI, 0.13 to 0.71)
logrank p = 0.004
HR = 1.19 (95% CI, 0.53 to 2.71)
logrank p = 0.67
Number at risk
lo 45 33 26 20 17 13 3
hi 40 36 34 30 29 27 3
lo 57 51 49 44 40 35 4
hi 52 49 43 39 37 33 3
C
Basal–like and HER2–enriched
Luminal A and B
VEGFA:CD8A
lo:lo
lo:hi
hi:lo
hi:hi
logrank p = 0.002
logrank p = 0.312
Number at risk
lo:lo 14 12 10 7 6 4 1
lo:hi 29 27 26 24 23 22 3
hi:lo 31 21 16 13 11 9 2
hi:hi 11 9 8 6 6 5 0
lo:lo 24 22 20 19 16 14 2
lo:hi 28 26 23 22 21 17 2
hi:lo 33 29 29 25 24 21 2
hi:hi 24 23 20 17 16 16 1

Figure S4, related to Figure 5.
(A-C) Association of *VEGFA* and *CD8A* expression with clinical outcome in the Uppsala cohort. Kaplan-Meyer survival curves of breast-cancer specific survival are shown for the indicated subtypes, for patients classified according to *VEGFA* relative expression levels (A), *CD8A* relative expression levels (B), and different ratios of *VEGFA*:*CD8A* relative expression levels (C).

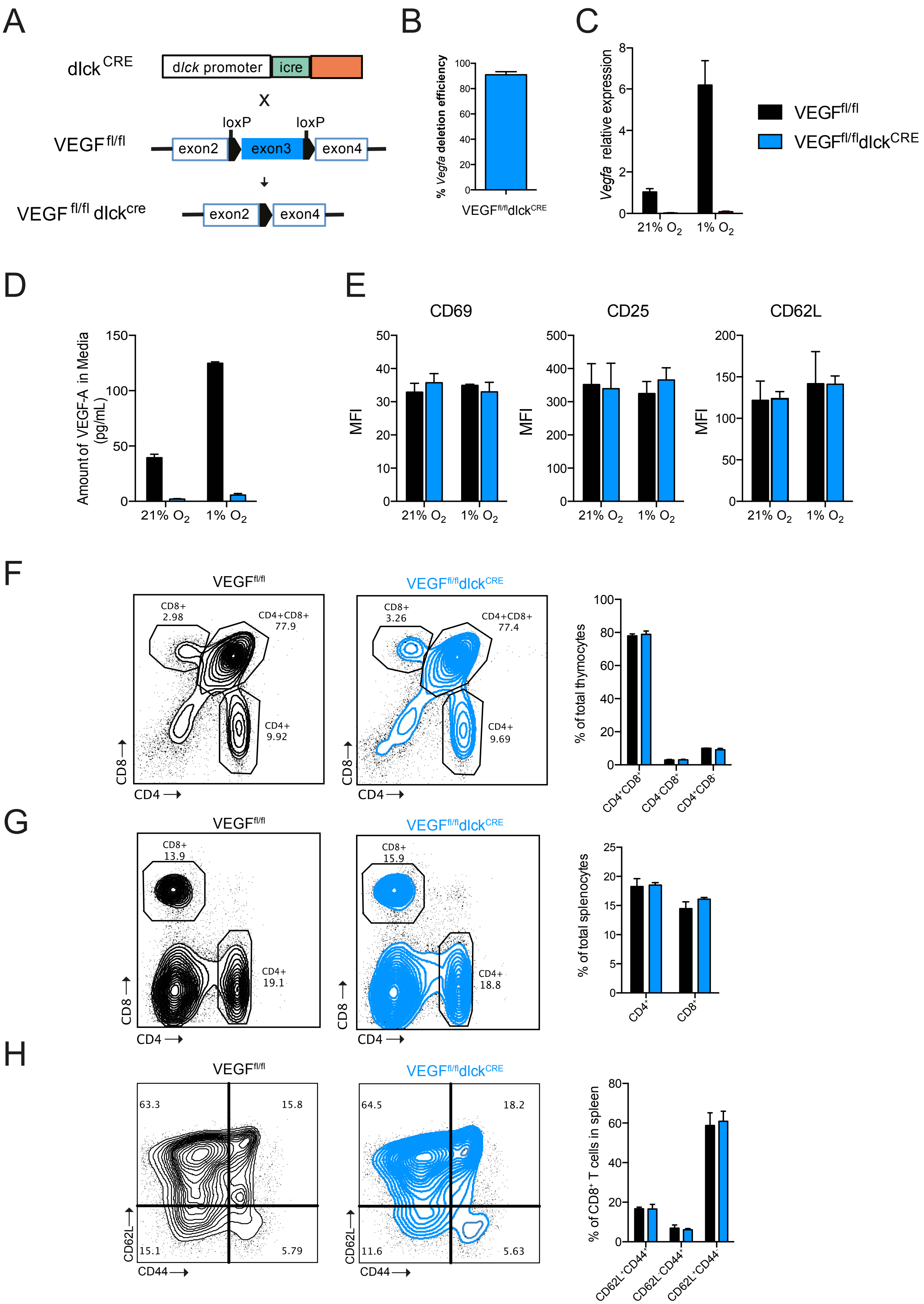
A
dlck CRE
dlck promoter
icre
x
loxP
loxP
VEGF fl/fl
exon2
exon3
exon4
VEGF fl/fl dlckcre
exon2
exon4
B
% Vegfa deletion efficiency
VEGFfl/fldlckCRE
C
Vegfa relative expression
21% O2
1% O2
VEGFfl/fl
VEGFfl/fldlckCRE
D
Amount of VEGF-A in Media (pg/mL)
E
CD69
CD25
CD62L
MFI
F
VEGFfl/fl
VEGFfl/fldlckCRE
CD8+
2.98
CD4+CD8+
77.9
CD4+
9.92
CD8+
3.26
CD4+CD8+
77.4
CD4+
9.69
CD8
CD4
% of total thymocytes
G
CD8+
13.9
CD4+
19.1
CD8+
15.9
CD4+
18.8
% of total splenocytes
H
63.3
15.8
15.1
5.79
64.5
18.2
11.6
5.63
CD62L
CD44
% of CD8+ T cells in spleen

Figure S5, related to Figure 6.

(A) Schematic diagram and gene deletion strategy to generate VEGF$^{fl/fl}$dlck^{CRE} mice. (B) Percentage of *Vegfa* deletion efficiency in gDNA from CD8^{+} CTLs generated *in vitro* after isolation from from VEGF$^{fl/fl}$dlck^{CRE} mice (n=4, error bars represent SD). (C) Relative expression of *Vegfa* by QRT-PCR on CTLs from mutant and control mice subjected to 24 hr of culture under 21% O_2 or 1% O_2 (n=4, error bars represent SD). (D) Amount of VEGF-A in media of CD8^{+} CTLs cultured as in (C), (n=4, error bars represent SD). (E) Mean fluorescence intensity for the surface expression of CD69, CD25 and CD62L measured by flow cytometry on CD8^{+} T cells 48 hr after activation with αCD3/αCD28. (n=4, error bars represent SD). (F) Thymic composition of mutant mice (VEGF$^{fl/fl}$dlck^{CRE}) and control littermates (VEGF$^{fl/fl}$)(n=4, error bars represent SD). (G) Splenic composition of mutant mice (VEGF$^{fl/fl}$dlck^{CRE}) and control littermates (VEGF$^{fl/fl}$) (n=4, error bars represent SD). (H) CD8^{+} T cell composition in spleen of mutant mice (VEGF$^{fl/fl}$dlck^{CRE}) and control littermates (VEGF$^{fl/fl}$) according to CD62L and CD44 expression(n=4, error bars represent SD).

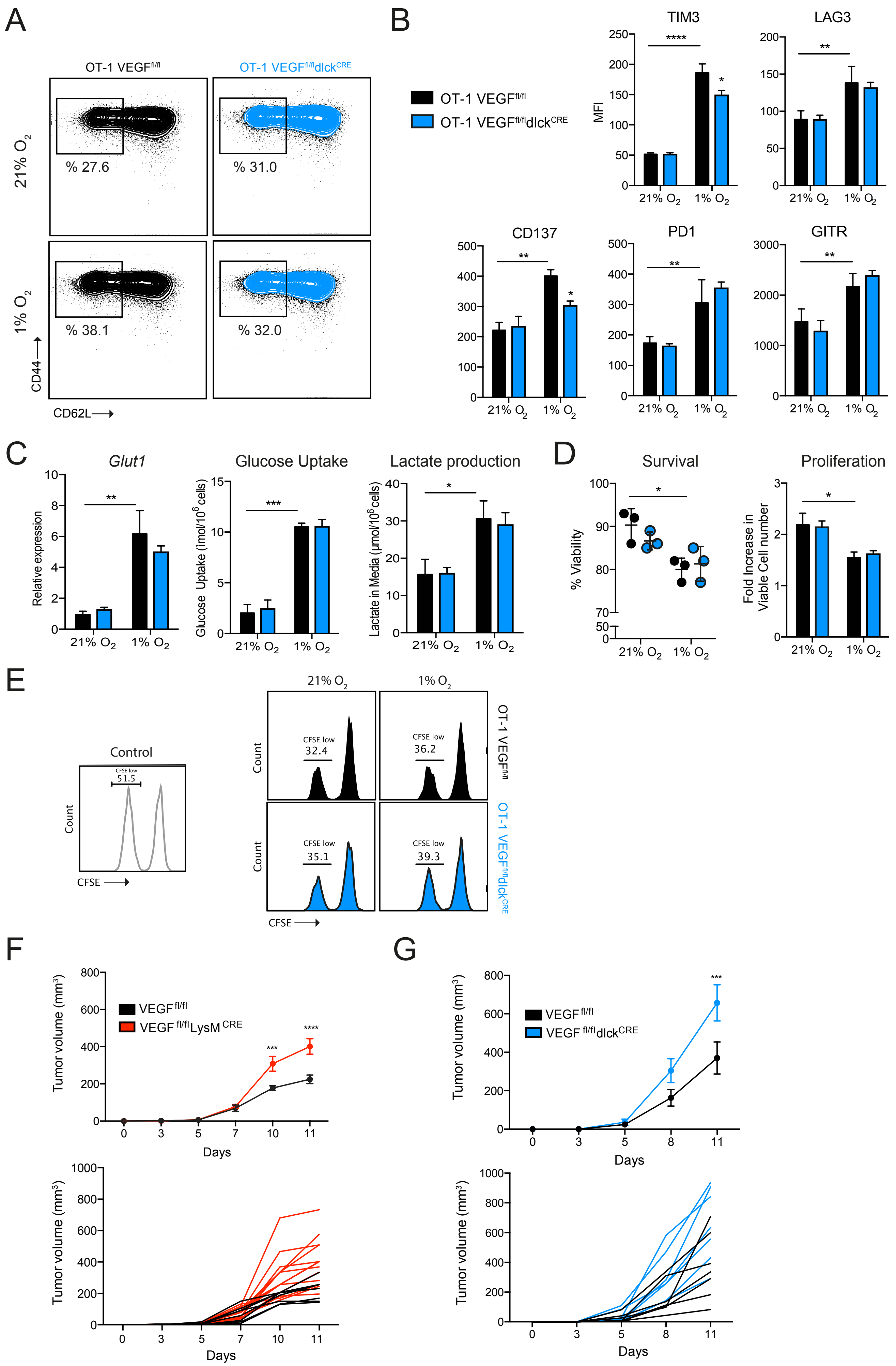
A
OT-1 VEGFfl/fl
OT-1 VEGFfl/fldlckCRE
21% O2
1% O2
% 27.6
% 31.0
% 38.1
% 32.0
CD44
CD62L
B
OT-1 VEGFfl/fl
OT-1 VEGFfl/fldlckCRE
TIM3
LAG3
CD137
PD1
GITR
MFI
21% O2
1% O2
C
Glut1
Glucose Uptake
Lactate production
Relative expression
Glucose Uptake (μmol/10^6 cells)
Lactate in Media (μmol/10^6 cells)
D
Survival
Proliferation
% Viability
Fold Increase in Viable Cell number
E
Control
CFSE low
51.5
32.4
36.2
35.1
39.3
Count
CFSE
OT-1 VEGFfl/fl
OT-1 VEGFfl/fldlckCRE
F
VEGFfl/fl
VEGF fl/flLysM CRE
Tumor volume (mm3)
Days
G
VEGFfl/fl
VEGF fl/fldlckCRE
Tumor volume (mm3)
Days

H

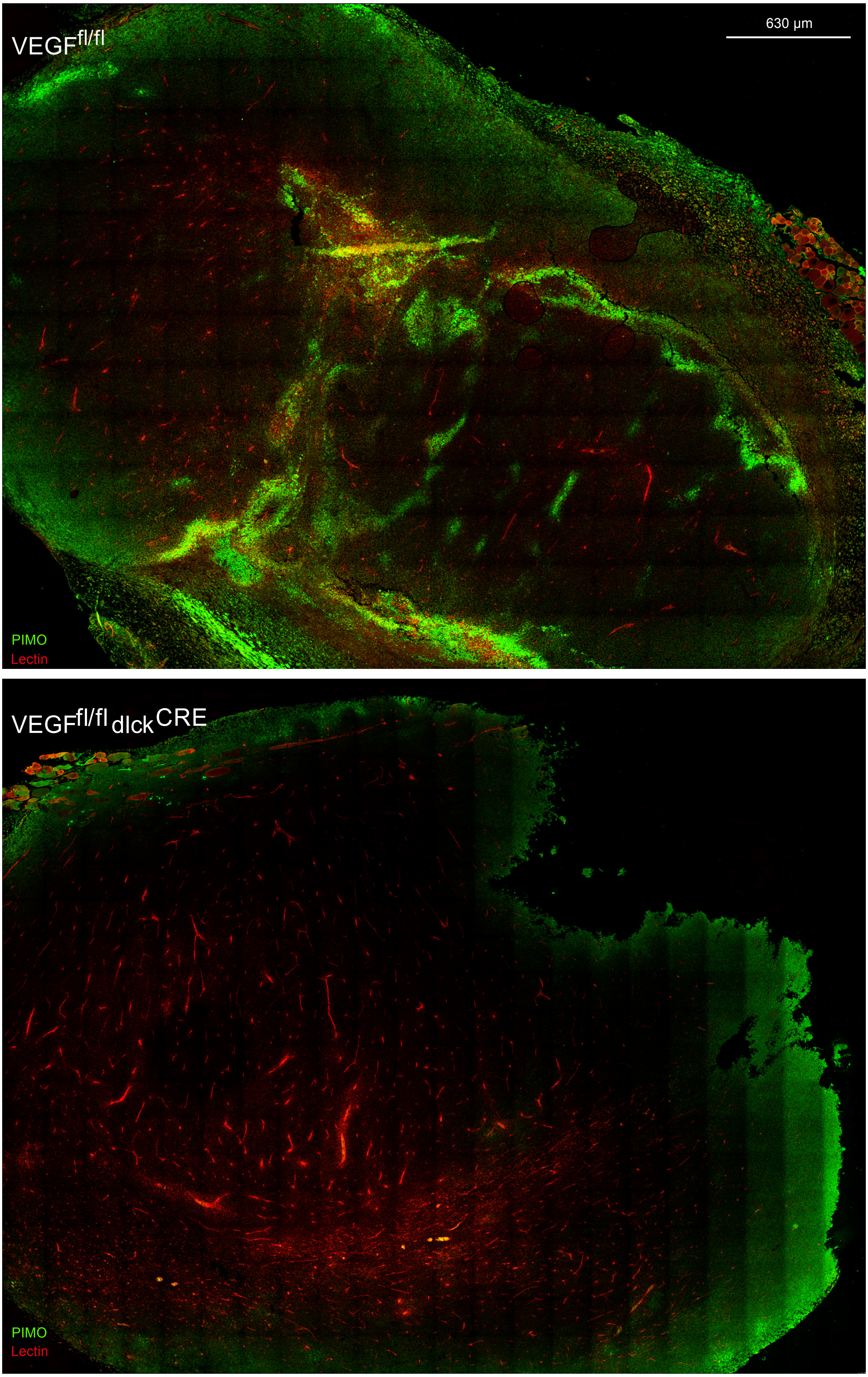

Figure S6, related to Figure 6

(A-D) Splenic $CD8^+$ T cells were isolated from mutant mice (OT-1 $VEGF^{fl/fl}dlck^{CRE}$) and control littermates (OT-1 $VEGF^{fl/fl}$), activated *in vitro* with cognate SIINFEKL peptide and expanded in the presence of IL-2 under 21% O_2 for 5 days and subjected to 21% O_2 or 1% O_2 for the last 24 hours: surface CD62L and CD44 expression by flow cytometry (A), surface expression of costimulatory/checkpoint receptors TIM3, LAG3, CD137, PD-1 and GITR (B), relative mRNA expression of Glut-1 on CTLs (C, Left), glucose uptake and lactate production by CTLs with the indicated genotypes (C, Right) and percent survival (D, Left) and proliferation (D, Right). (n=4, error bars represent SD). (E) *In vitro* killing assay: target cells were pulsed with peptide and stained with $CFSE^{lo}$ and mixed in equal proportions with unpulsed $CFSE^{hi}$ cells. CTLs from mutant or control mice were added to the culture for 4 hours and % of $CFSE^+$ cells were quantified by flow cytometry. (F-G) Representative growth curves of subcutaneously injected LLC tumors in $VEGF^{fl/fl}LysM^{CRE}$ (red, n=13) (F) and $VEGF^{fl/fl}dlck^{CRE}$ (blue, n=7) (G). $VEGF^{fl/fl}$ littermate controls are shown in black, ($0.5x10^6$ tumor cells/mouse). Error bars represent SEM, statistical analysis was performed by two-way ANOVA with Sidak correction for multiple comparisons. (H) Representative immunofluorescence images from LLC tumors for the indicated genotypes (Green= pimonidazole, Red= Tomato lectin-Dylight-594).

Table S2, related to STAR Methods. Primers for qRT-PCR.

Gene	Forward primer	Reverse primer
Rn18s	5'-CGGCGACGACCCATTCGAAC-3'	5'-GAATCGAACCCTGATTCCCCGTC-3'
Hprt	5’- TCAGTCAACGGGGGACATAAA-3’	5’- GGGGCTGTACTGCTTAACCAG-3’
Pgk1	5'-ATTCTGCTTGGACAATGGAGC-3'	5'-AGGCATGGGAACACCATCA-3'
Hk2	5′-TGATCGCCTGCTTATTCACGG-3′	5'-AACCGCCTAGAAATCTCCAGA-3'
Pdk1	5'-GAAGCAGTTCCTGGACTTCG-3'	5'-CCAACTTTGCACCAGCTGTA-3'
Vegfa	5'-CCACGTCAGAGAGCAACATCA-3'	5'-TCATTCTCTCTATGTGCTGGCTTT-3'
Mct4	5'-TCACGGGTTTCTCCTACGC-3'	5'-GCCAAAGCGGTTCACACAC-3'
Hif1a	5'-GAAACGACCACTGCTAAGGCA-3'	5'-GGCAGACAGCTTAAGGCTCCT-3'
Epas1	5'-CAACCTGCAGCCTCAGTGT-3'	5'-CACCACGTCGTTCTTCTCGA-3'

Table S3, related to STAR Methods. Primers for determining deletion efficiency.

Actb Forward	5’-AGAGGGAAATCGTGCGTGA-3’
Actb Reverse	5’-CAATAGTGATGACCTGGCCGT-3’
Actb Probe	5’-[6-FAM] CACTGCCGCATCCTCTTCCTC [BHQ1a-Q]-3’
Vegfa Forward	5'-TGACCATCTGCTTTCGTGACC-3’
Vegfa Reverse	5'-ACTTGTTGCAGGCAGCGG-3’
Vegfa Probe	5’-[6-FAM] TGCTCCCTGGGCTCGACAGGG [BHQ1a-Q]-3’
Hif1a Forward	5’ GGTGCTGGTGTCCAAAATGTAG 3’
Hif1a Reverse	5’ ATGGGTCTAGAGAGATAGCTCCACA 3’
Hif1a Probe	5’ [6-FAM] CCTGTTGGTTGCGCAGCAAGCATT [BHQ1a-Q] 3’
Hif2a Forward	5’ TCTATGAGTTGGCTCATGAGTTG 3’
Hif2a Reverse	5’ GTCCGAAGGAAGCTGATGG 3’
Hif2a Probe	5’ [6-FAM] CCACCTGGA/ZEN/CAAAGCCTCCATCAT [3IABkFQ] 3’
