# Supplementary material for: An HIF-1α/VEGF-A Axis in Cytotoxic T Cells Regulates Tumor Progression
Source: Cancer Cell. 2017 Nov 13;32(5):669–683.e5. doi: 10.1016/j.ccell.2017.10.003 (PMC5691891; doi:10.1016/j.ccell.2017.10.003)
Supplement: Document S2. Article plus Supplemental Information [file mmc2.pdf]

Article

Cancer Cell

An HIF-1α/VEGF-A Axis in Cytotoxic T Cells Regulates Tumor Progression

Highlights

- HIF-1α drives CD8$^+$ T cell migration and effector function
- HIF-1α loss in T cells accelerates tumor growth
- Loss of VEGF-A in T cells accelerates tumor growth
- Loss of VEGF-A in T cells results in increased chemotherapeutic response

Authors

Asis Palazon, Petros A. Tyrakis, David Macias, ..., Ananda W. Goldrath, Jonas Bergh, Randall S. Johnson

Correspondence

rsj33@cam.ac.uk

In Brief

Palazon et al. demonstrate the importance of the HIF-1α/VEGF-A axis in tumor immunity. HIF-1α, but not HIF-2α, drives CD8$^+$ T cell glycolytic metabolism, migration, and effector function, while the HIF-1α transcriptional target VEGF-A contributes to tumor vascularization.

Palazon et al., 2017, Cancer Cell *32*, 669–683
November 13, 2017 © 2017 The Authors. Published by Elsevier Inc.
https://doi.org/10.1016/j.ccell.2017.10.003

CellPress

An HIF-1α/VEGF-A Axis in Cytotoxic T Cells Regulates Tumor Progression

Asis Palazon,[1] Petros A. Tyrakis,[1,2] David Macias,[2] Pedro Veliça,[3] Helene Rundqvist,[3] Susan Fitzpatrick,[2] Nikola Vojnovic,[3] Anthony T. Phan,[4] Niklas Loman,[6] Ingrid Hedenfalk,[6] Thomas Hatschek,[5] John Lövrot,[5] Theodoros Foukakis,[5] Ananda W. Goldrath,[4] Jonas Bergh,[5] and Randall S. Johnson[1,3,*]

[1]Department of Physiology, Development and Neuroscience, University of Cambridge, Cambridge CB2 3EG, UK
[2]Cancer Research UK, Cambridge Institute, Cambridge CB2 0RE, UK
[3]Department of Cell and Molecular Biology, Karolinska Institute, 171 77 Stockholm, Sweden
[4]Molecular Biology Section, Division of Biological Sciences, University of California San Diego, La Jolla, CA 92161, USA
[5]Karolinska Oncology, Karolinska Institute and University Hospital, 171 76 Stockholm, Sweden
[6]Department of Clinical Sciences, Division of Oncology and Pathology, Lund University, 223 81 Lund, Sweden
*Correspondence: rsj33@cam.ac.uk
https://doi.org/10.1016/j.ccell.2017.10.003

SUMMARY

Cytotoxic T cells infiltrating tumors are thought to utilize HIF transcription factors during adaptation to the hypoxic tumor microenvironment. Deletion analyses of the two key HIF isoforms found that HIF-1α, but not HIF-2α, was essential for the effector state in $CD8^+$ T cells. Furthermore, loss of HIF-1α in $CD8^+$ T cells reduced tumor infiltration and tumor cell killing, and altered tumor vascularization. Deletion of VEGF-A, an HIF target gene, in $CD8^+$ T cells accelerated tumorigenesis while also altering vascularization. Analyses of human breast cancer showed inverse correlations between VEGF-A expression and $CD8^+$ T cell infiltration, and a link between T cell infiltration and vascularization. These data demonstrate that the HIF-1α/VEGF-A axis is an essential aspect of tumor immunity.

INTRODUCTION

Hypoxia-inducible transcription factors (HIFs) have a central role in physiological adaptation to varying oxygenation states. They act as essential controls for adaptive changes at the cellular, tissue, and organismal levels in response to a range of challenges, including altered tissue oxygenation (Semenza, 2012). Two HIF transcription factors comprise the best characterized elements of this response: both are oxygen sensitive and in both cases their activity can be regulated post-transcriptionally, via the von Hippel-Lindau (VHL) tumor-suppressor complex and the FIH asparagine hydroxylase. The first element of the pathway to be cloned was the HIF-1α factor; it is expressed in most if not all mammalian cell types. It has important functional roles in both innate and adaptive immune cells, including macrophages (Cramer et al., 2003), neutrophils (Walmsley et al., 2005), dendritic cells (Jantsch et al., 2008), and lymphocytes (McNamee et al., 2013). A second well-characterized element of the HIF pathway is HIF-2α: its functional role includes regulation of erythropoiesis. The role of HIF-2α in the immune system is less clear, but appears to be important for the regulation of nitric oxide homeostasis in innate cells and controlling arginase expression, among its other roles (Takeda et al., 2010).

It has become clear that the HIF transcription factors are regulated in a complex fashion and are controlled by many factors other than oxygenation. In the immune system cytokines can induce both HIF-1α and HIF-2α in response to a range of challenges, including inflammation and infection (reviewed in Palazon et al., 2014). In T lymphocytes, HIF-1α is stabilized upon T cell receptor (TCR) activation (Nakamura et al., 2005) and is important for a metabolic transition to glycolysis, which in turn supports proliferation and effector function (Buck et al., 2015; Pearce et al., 2013).

Significance

Cytotoxic T lymphocytes respond to the immune microenvironment and tissue oxygenation. That response in turn is regulated in significant part by the hypoxia-inducible transcription factor (HIF). Here we show that that the HIF-1α/VEGF-A response in T cells has direct effects on cancer growth, progression, and vascularization, suggesting that therapeutic strategies targeting HIF-1α or VEGF-A signaling in the tumor microenvironment will affect cancer therapy generally and immunotherapy specifically. These findings also show that modulating the hypoxic response in cytotoxic T cells has striking effects on tumor vascularization and progression; exploitation of this knowledge could significantly advance immunotherapeutic approaches.

This is an open access article under the CC BY license (http://creativecommons.org/licenses/by/4.0/).

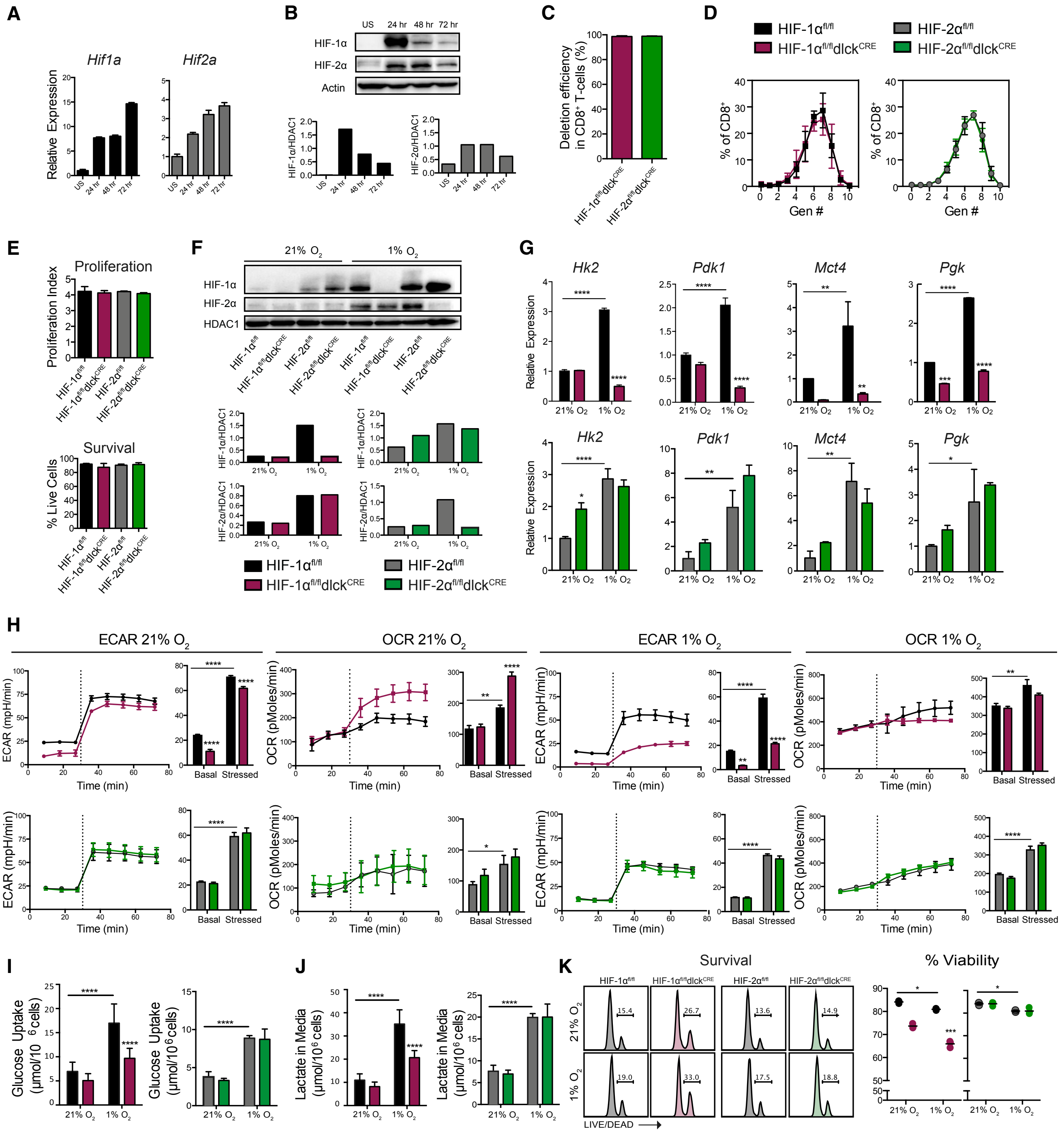

Figure 1. Hypoxia Promotes CD8$^+$ T Cell Glycolytic Metabolism in an HIF-1α- but Not HIF-2α-Dependent Fashion

(A) qRT-PCR of mRNA levels of *Hif1a* and *Hif2a* on magnetically isolated splenic CD8$^+$ T cells before and after activation with αCD3/CD28 for the indicated time points (n = 3). US, unstimulated. Error bars represent SD.

(B) Immunoblots showing HIF-1α and HIF-2α expression in T cells collected at the indicated time points after activation. Densitometric analyses are shown at the bottom.

(C) Deletion efficiency of *Hif1a* and *Hif2a* in genomic DNA from CD8$^+$ lymphocytes isolated from HIF-1α$^{fl/fl}$dlck^{CRE} or HIF-2α$^{fl/fl}$dlck^{CRE} mice (n = 4, error bars represent SD).

(D) CFSE (carboxyfluorescein succinimidyl ester) dilution assay 72 hr after *in vitro* activation (n = 3, error bars represent SD).

(E) Proliferation index and percent survival of isolated CD8$^+$ T lymphocytes 4 days after activation (n = 4, error bars represent SD).

(F) CD8$^+$ T cells were isolated from spleens and activated with αCD3/CD28 for 48 hr, and then expanded for 3 days in IL-2 and subjected to 21% or 1% O_2 for 24 hr. Western blotting was performed on nuclear fractions; densitometric analyses are shown.

(legend continued on next page)

Deletion of the aryl hydrocarbon nuclear translocator (ARNT)/HIF-1β gene in CD8$^+$ T cells impairs the expression of cytolytic effector molecules, including perforins and granzymes, and alters the expression of CD62L and cytokines and cytokine receptors involved in the migration of activated T cells from secondary lymphoid organs to peripheral tissues (Finlay et al., 2012). Although the *Arnt* deletion has a range of effects, blocking the action of both the HIF-α isoforms, as well as the aryl hydrocarbon receptor (AhR), these experiments illustrate how important the role of the HIF pathway in T cell function might be. Further evidence for HIF's role in adaptive immunity came from experiments utilizing mice with a deletion of *Vhl* in CD8$^+$ T cells. This deficiency of a key negative regulator of HIF caused increased expression of both HIF-α isoforms and a resultant increase in glycolytic activity, as well as increased expression of costimulatory receptors and cytolytic molecules. This in turn led to increased effector function (Doedens et al., 2013).

The deletion of *Vhl* in T cells results in increased anti-tumor activity in a T cell-dependent model of tumor killing. Loss of VHL removes certain aspects of oxygen-regulated control of expression of the HIF transcription factors; however, as noted above, there is considerable complexity to the pathway. It is also unclear which of the numerous targets of HIF function might play an important role in T cell function in the tumor microenvironment.

Arguably, the most well-studied HIF target gene is the angiogenic/permeability vascular endothelial growth factor A (VEGF-A), which is expressed in both tumor and stromal cells. Although VEGF-A production by tumor cells has been correlated with poor prognosis, pharmacological VEGF-A blockade has shown limited therapeutic success; one likely reason is that VEGF-A from non-endothelial stromal populations can enable tumor survival (Shojaei et al., 2008). In previous studies, we genetically deleted *Vegfa* in myeloid cells (Stockmann et al., 2008) and fibroblasts (Kim et al., 2012); in both cases this led to accelerated tumor growth. Tumor-infiltrating lymphocytes secrete VEGF-A (Freeman et al., 1995); however, the contribution of T cell-derived VEGF-A to lymphocyte function and tumor progression is not clear.

RESULTS

Hypoxia Affects CD8$^+$ T Cell Glycolytic Metabolism in an HIF-1α- but Not HIF-2α-Dependent Fashion

Hypoxia induces a shift toward an anaerobic and glycolytic metabolism, and HIF function is associated with the regulation of glycolysis (Seagroves et al., 2001) and the shift to a suppression of oxidative metabolism (Kim et al., 2006; Papandreou et al., 2006). T cell activation and proliferation are themselves correlated with increased glycolysis (Buck et al., 2015). As can be seen, TCR stimulation results in increases in *Hif1a* and *Hif2a* mRNA expression (Figure 1A) and protein accumulation (Figure 1B). Additionally, both the normal tissue environment and tumors have levels of oxygen that will activate the HIF pathway, which is thought to become highly active at levels corresponding to less than 5% oxygen in tissue culture systems (Semenza, 2012); we found by oxygen electrode measurements that murine spleens have a mean pO_2 of approximately 22 mmHg, which would approximate a tissue culture environmental oxygen level of approximately 3% (Table S1). Solid tumors are known to have much lower pO_2 values, ranging well below 10 mmHg (Vaupel et al., 1989). Thus, HIF response will affect T cells in both normal and malignant tissues.

To study the effects of HIF-1α and HIF-2α in CD8$^+$ T cells, we genetically ablated exon2 from the *Hif1a* (Ryan et al., 2000) or *Epas* alleles (Gruber et al., 2007) by a Cre-recombinase expressed under the control of the distal promoter of the lymphocyte protein tyrosine kinase gene (dlck) (HIF-1α$^{fl/fl}$dlck^{CRE} and HIF-2α$^{fl/fl}$dlck^{CRE}); this transgene is expressed at a late stage of T cell development (Zhang et al., 2005; Zhang and Bevan, 2010). This strategy results in a complete deletion of *Hif1a* and *Hif2a* in CD8$^+$ T cells (Figure 1C) and does not affect T cell proliferation or survival at 72 hr after activation in 21% oxygen conditions (Figures 1D and 1E). Deletion in CD4$^+$ T cells in this model occurs to a lesser extent (Zhang et al., 2005) (Figure S1). We cultured mutant and control cytotoxic T lymphocytes (CTLs) under normoxic (21% O_2) and hypoxic (1% O_2) conditions (Figure 1F). As CD8$^+$ T cells differentiated into effectors, CD8$^+$ T cells lacking HIF-1α, but not HIF-2α, showed impaired expression of genes involved in glycolytic metabolism (Figure 1G). HIF-1α plays an essential role in the maintenance of glycolysis, as shown by extracellular flux analyses (Figure 1H), and glucose uptake and lactate production (Figures 1I and 1J). CD8$^+$ T cells lacking HIF-2α have a normal glycolytic metabolism and metabolic shift during hypoxic treatment (Figures 1H–1J). Hypoxia and loss of HIF-1α, but not HIF-2α, modestly decreased the survival of terminally differentiated CD8$^+$ CTLs (Figure 1K).

Hypoxia and HIF-1α Supports T Cell Acquisition of Effector Function

HIF-1α-, but not HIF-2α-deficient effector CD8$^+$ T cells failed to downregulate the cell-surface expression of CD62L (Figure 2A), a factor associated with T cell activation and migration of CD44$^+$ antigen-experienced cells following *in vitro* activation and expansion in the presence of interleukin-2 (IL-2). Additionally, HIF-1α ablation significantly decreased the production of

(G) CD8$^+$ T cells from HIF-1α$^{fl/fl}$dlck^{CRE} (maroon), HIF-2α$^{fl/fl}$dlck^{CRE} (green), and littermate controls (black for HIF-1α$^{fl/fl}$, gray for HIF-2α$^{fl/fl}$) were isolated, activated, expanded for 5 days in the presence of IL-2, and cultured for 24 hr under 21% versus 1% O_2. qRT-PCR was performed for *Hk2*, *Pdk1*, *Mct4*, and *Pgk* (n = 3, error bars represent SD).

(H) Extracellular acidification rate (ECAR) and oxygen consumption rate (OCR) of CD8$^+$ T cells prepared as in (G) were measured by flux analysis, under basal conditions and after injection (dashed line) of oligomycin and FCCP (stressed) (n = 4 per genotype, error bars represent SEM).

(I and J) Supernatants of CD8$^+$ T cells cultured as in (G) were measured for glucose (I) and lactate (J) levels (n = 6, error bars represent SD).

(K) Representative flow-cytometry histograms of viability staining and percent survival of CD8$^+$ T cells cultured as in (G) (n = 4). Grouped data were assessed by two-way ANOVA for multiple comparisons with Bonferroni correction.

Horizontal lines are the mean values. ****$p < 0.00005$, ***$p < 0.0005$, **$p < 0.005$, *$p < 0.05$. See also Table S1 and Figure S1.

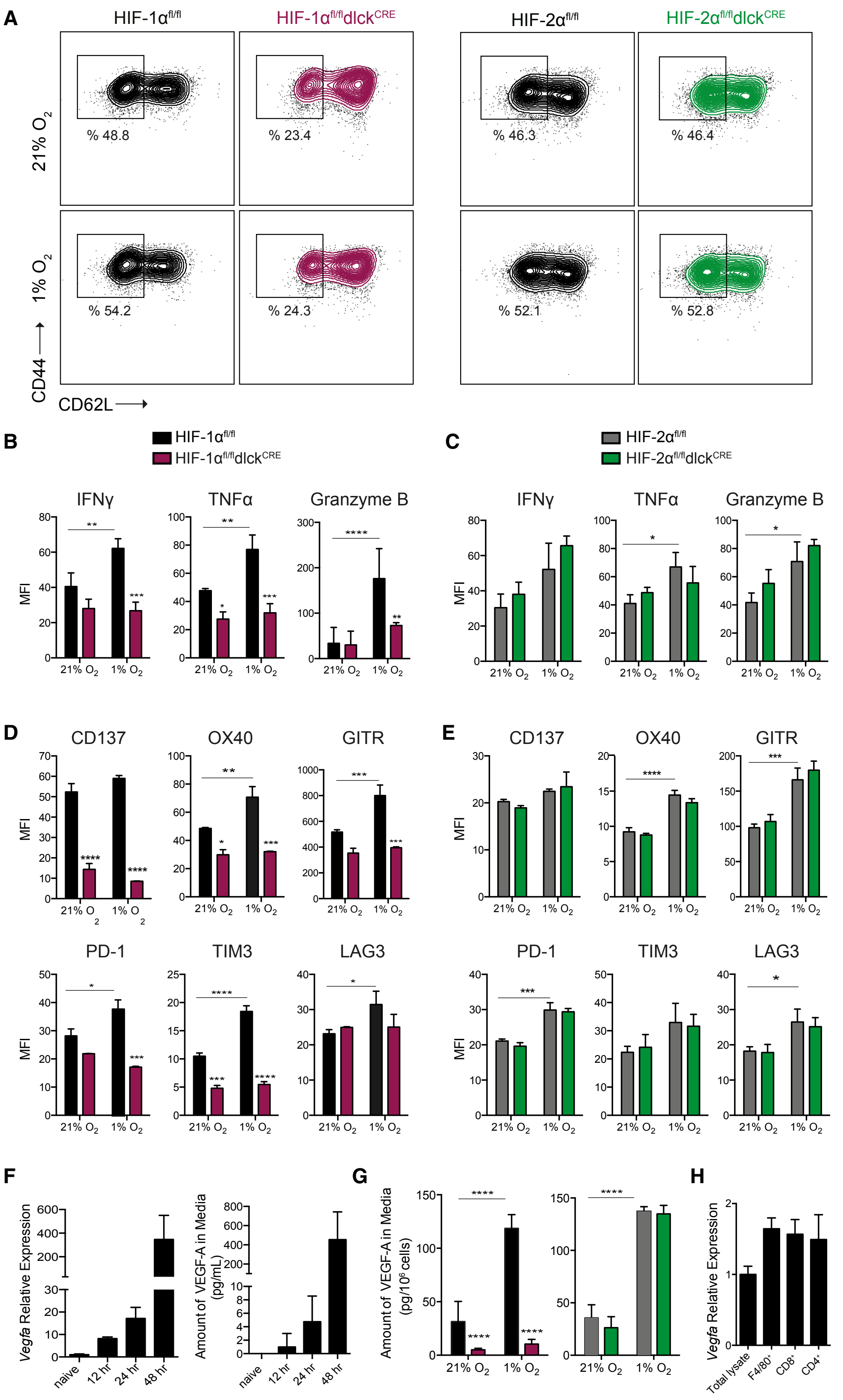

(legend on next page)

the effector cytokines interferon γ (IFNγ) and tumor necrosis factor α (TNFα) by CTLs (Figure 2B). Hypoxia increased the production of the cytolytic molecule granzyme B (Figure 2B) and the expression of the activation-related costimulatory molecules CD137, OX40, and GITR, and checkpoint receptors PD-1, TIM3, and LAG3. These changes occurred in an HIF-1α-, but not HIF-2α-dependent manner following *in vitro* activation and addition of IL-2 (Figures 2B–2E). This is in line with our previous findings supporting a model in which HIFα isoform usage and the targets regulated are dependent on cytokine milieu. Interestingly, $CD8^+$ T cells lacking either both VHL and HIF-1α or both HIF-1α and HIF-2α failed to downregulate surface expression of CD62L and acquire an effector phenotype after activation (Figures S2A–S2H). These defects had an impact on the cytotoxic capacity of antigen-specific HIF-mutant $CD8^+$ T cells, which showed a reduced ability to kill target cells *in vitro* (Figure S2I).

As can be seen in Figure 2F, VEGF-A production increased with T cell activation, and T cell culture under 1% O_2 conditions further increased VEGF-A secretion. This expression was shown by deletion analysis to be driven by HIF-1α, but not HIF-2α, following expansion in IL-2 (Figure 2G). T cell-derived *Vegfa* is likely a significant source of the growth factor in immune infiltrates; Figure 2H shows that T cells infiltrating Lewis lung carcinoma (LLC) tumors express *Vegfa* to a level comparable with the levels of expression in F4/80^+ macrophages.

Genetic Deletion of HIF-1α in T Lymphocytes Leads to Accelerated Tumor Growth Characterized by Impaired $CD8^+$ T Cell Infiltration

To determine the role played by the HIF pathway in polyclonal $CD8^+$ T cells during tumor growth, we subcutaneously grafted LLC and B16-F10 melanoma cells into mice specifically lacking HIF-1α or HIF-2α in T lymphocytes (HIF-1$\alpha^{fl/fl}$dlck^{CRE} or HIF-2$\alpha^{fl/fl}$dlck^{CRE}), and compared tumor growth with that seen in wild-type (WT) control littermates (HIF-1$\alpha^{fl/fl}$ and HIF-2$\alpha^{fl/fl}$). Tumors growing in mice lacking HIF-1α in T cells expanded more rapidly (Figures 3A and 3B) and were significantly larger at endpoint (Figure 3C). This was not true for tumors grown in HIF-2α-deficient animals.

The composition of tumor-infiltrating lymphocytes in these experimental tumors was lower in $CD8^+$ T cells in mice deficient in HIF-1α in T cells, and there was a diminished $CD8^+$-to-$CD4^+$ T cell ratio (Figure 3D). HIF-1α-deficient tumor-infiltrating $CD8^+$ lymphocytes were characterized by a drop in expression of the costimulatory molecules CD137 and GITR, and in the checkpoint receptors PD-1, LAG3, and TIM3 (Figure 3E). The HIF-1α-dependent reduction in PD-1 seen in tumor-infiltrating lymphocytes is surprising given that previous work in chronic viral infection found PD-1 expression to be HIFα independent, but this result may reflect model-specific differences, such as inflammatory milieu, between tumor and systemic chronic viral infections (Doedens et al., 2013).

Moreover, FOXP3-expressing $CD4^+$ T cells were enriched in the mutant mice, leading to a decreased IFNγ^+/FOXP3^+ ratio (Figure 3F). This is consistent with recent reports highlighting the role of HIF-1α in the promotion of IFNγ production by $CD4^+$ T cells (Lee et al., 2015), and the regulation of FOXP3 degradation by HIF-1α (Dang et al., 2011).

As described above, there are lower numbers of $CD8^+$ T cells in tumors from animals where T cells lack HIF-1α. This may be in part related to a deficient capacity to traverse endothelial barriers. To determine how HIF-1α expression in $CD8^+$ T cells affects migration through endothelium, we performed *in vitro* coculture experiments in which activated primary WT control or mutant $CD8^+$ T cells were isolated and cultured in hypoxic conditions in a Boyden chamber. The Boyden chamber used for these experiments was lined with early-passage murine primary WT pulmonary endothelial cells. Under these conditions $CD8^+$ T cells lacking HIF-1α have a significantly reduced rate of migration, with less than half as many mutant T cells on the other side of the barrier relative to the number of WT T cells after 24 hr of culture under hypoxia (Figure 3G).

To further characterize tumor vascularization in animals with HIF-mutant T cells, we injected mutant and control LLC tumor-bearing mice with fluorescence-conjugated tomato lectin. Tumor vascularization was then characterized by image detection algorithms, which showed that tumors in mutant mice differed significantly in the length of vascular segments between branch points, had altered vascular architecture (vessel straightness), and showed a trend to an increase in the overall amount of patent endothelium (Figures 3H and 3I). Increased vessel length

Figure 2. Hypoxia and HIF-1α, but Not HIF-2α, Support $CD8^+$ T Cell Acquisition of Effector Phenotype

(A) $CD8^+$ T cells were isolated from spleens of HIF-1$\alpha^{fl/fl}$dlck^{CRE} (maroon) or HIF-2$\alpha^{fl/fl}$dlck^{CRE} (green) and littermate control (black) mice, and activated with αCD3/CD28 for 48 hr, then expanded for 5 days in IL-2 and subjected to 21% or 1% O_2 for 24 hr. Expression of CD44 and CD62L by flow cytometry is shown.

(B) Intracellular expression of IFNγ, TNFα, and granzyme B in HIF-1$\alpha^{fl/fl}$dlck^{CRE} (maroon) and HIF-1$\alpha^{fl/fl}$ control littermates (black) by flow cytometry after restimulation (n = 3, error bars represent SD).

(C) Intracellular expression of IFNγ, TNFα, and granzyme B in HIF-2$\alpha^{fl/fl}$dlck^{CRE} (green) and HIF-2$\alpha^{fl/fl}$ control littermates (gray) by flow cytometry after restimulation (n = 3, error bars represent SD).

(D) Surface expression of costimulatory molecules/checkpoint receptors CD137, OX40, GITR, PD-1, TIM-3, and LAG3 on $CD8^+$ T cells isolated from HIF-1$\alpha^{fl/fl}$dlck^{CRE} (maroon) HIF-1$\alpha^{fl/fl}$ control littermates (black), activated and expanded as in (A) (n = 3, error bars represent SD).

(E) Same as in (D) on $CD8^+$ T cells isolated from HIF-2$\alpha^{fl/fl}$dlck^{CRE} (green) or HIF-2$\alpha^{fl/fl}$ control littermate mice (gray) (n = 3, error bars represent SD).

(F) Relative mRNA levels of *Vegfa* (left, n = 3) and amount of VEGF-A in media (right, n = 4, error bars represent SD) on $CD8^+$ T cells for the indicated time points after αCD3/CD28 activation.

(G) Amount of VEGF-A in media from CTLs expanded as in (A) and cultured under 21% O_2 or 1% O_2 for 24 hr (n = 4, error bars represent SD).

(H) Relative expression of *Vegfa* in total LLC tumor lysate and immune populations isolated from subcutaneous Lewis lung carcinoma (LLC) tumors 11 days after implantation (n = 10, error bars represent SD).

Grouped data were assessed by two-way ANOVA for multiple comparisons with Bonferroni correction. ****$p < 0.00005$, ***$p < 0.0005$, **$p < 0.005$, *$p < 0.05$. MFI, mean fluorescence intensity. See also Figure S2.

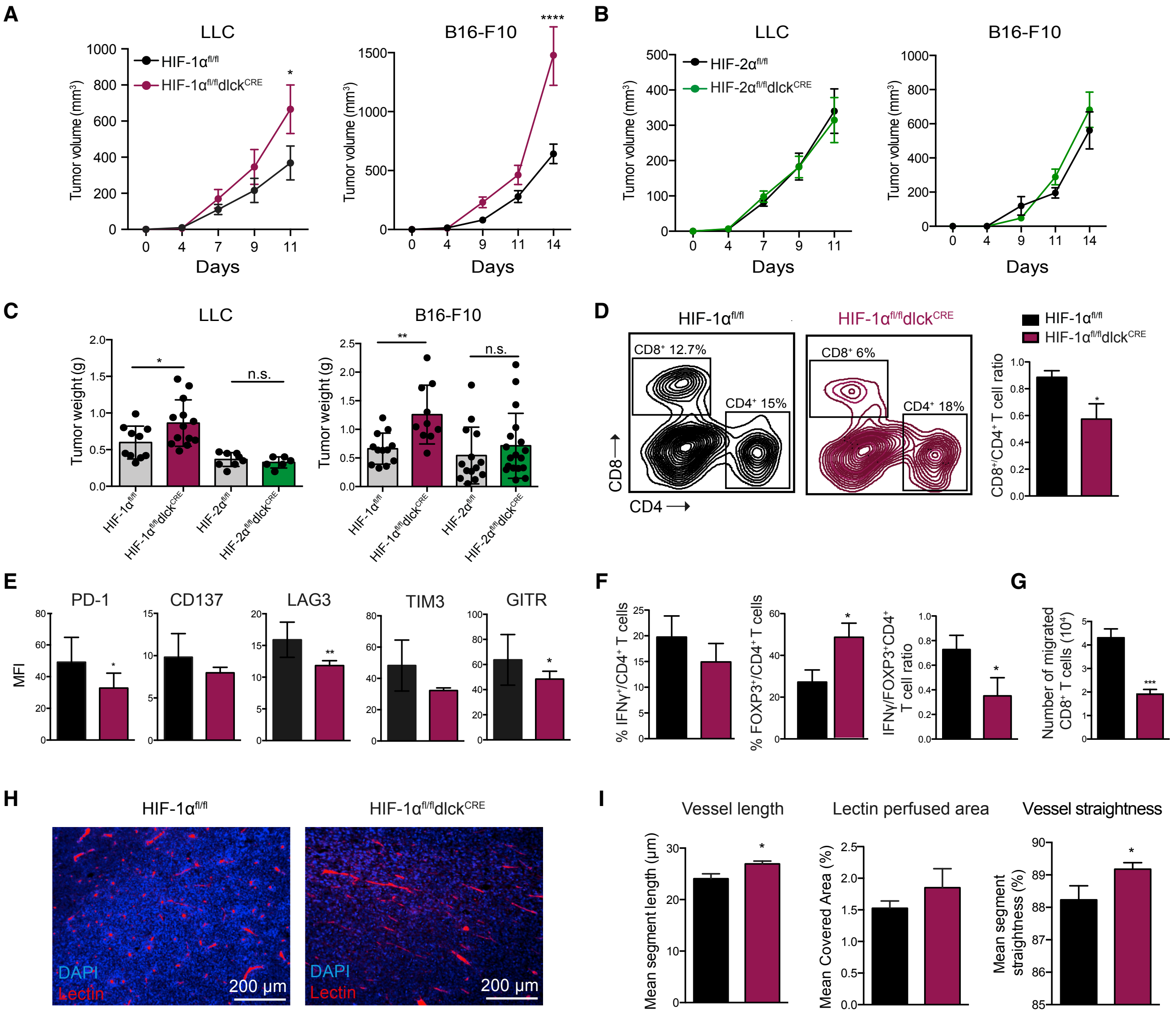

Figure 3. Genetic Deletion of HIF-1α, but Not HIF-2α, in T Lymphocytes Leads to Accelerated Tumor Growth and Impaired CD8+ T Cell Tumor Infiltration

(A and B) Growth curves of subcutaneous LLC and B16-F10 tumors (0.5×10^6 cells/mouse) in HIF-1α mutant mice (maroon) and littermate controls (black) (A, n = 10–13 per group) or HIF-2α mutant mice (green) versus littermate controls (black) (B, n = 6–17 per group). Statistical analysis was performed by two-way ANOVA with Sidak correction for multiple comparisons; tumor volumes are shown (mean values ± SEM).

(C) Tumor weights at the endpoint of the experiment (error bars represent SD).

(D) Representative dot plots showing $CD8^+$ and $CD4^+$ T cell tumor infiltration in mutant mice and littermate controls by flow cytometry (left) and $CD8^+$ to $CD4^+$ ratio in TILs (right, n = 6, error bars represent SD).

(E) Expression of costimulatory receptors and checkpoint inhibitors on $CD8^+$ T cells infiltrating LLC isografts 13 days after subcutaneous inoculation (n = 6, error bars represent SD).

(F) Flow-cytometry analyses on $CD4^+$ TILs: $IFN\gamma^+$ cells, percent $FOXP3^+$ cells, and $IFN\gamma^+/FOXP3^+$ $CD4^+$ T cell ratio (n = 6, error bars represent SD).

(G) HIF-1α-deficient (maroon) and control (black) $CD8^+$ T cells were subjected to 1% O_2 and cultured in a Boyden chamber on top of a confluent layer of primary endothelial cells. Twelve hours later T cells in the bottom well were counted (n = 4, error bars represent SD).

(H) Immunofluorescence images from LLC tumors for the indicated genotypes (blue = DAPI, red = tomato lectin-Dylight-594).

(I) Analyses of tomato lectin-Dylight-594-stained LLC tumor sections: mean vessel length, percent covered area, and vessel straightness. The means of these parameters per mouse are shown (6 fields per mouse, n ≥ 6 mice per group, error bars represent SEM).

****$p < 0.00005$, ***$p < 0.0005$, **$p < 0.005$, *$p < 0.05$; n.s., not significant.

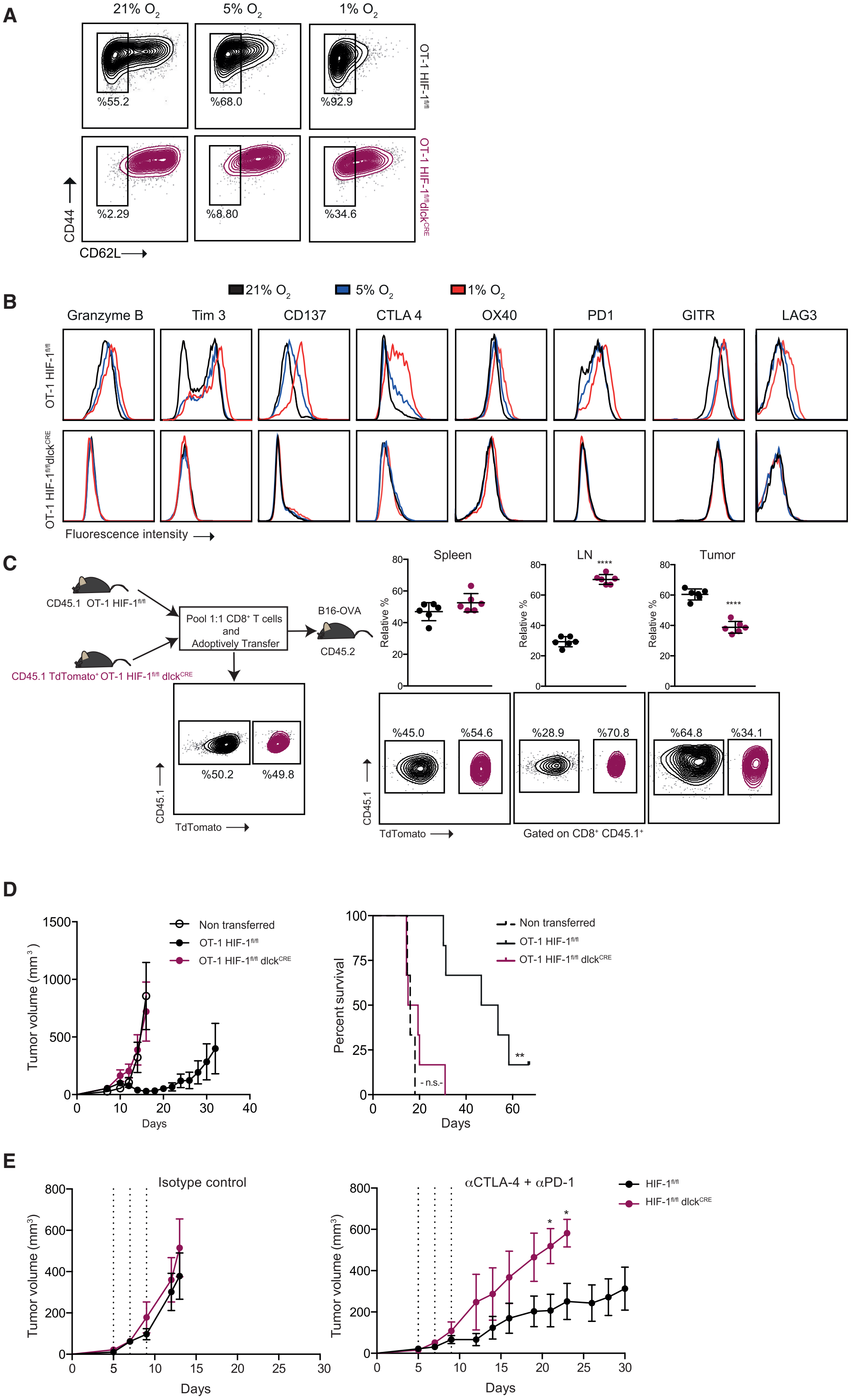

(legend on next page)

and tumor perfusion and decreased tortuosity are hallmarks of vascular normalization.

Genetic Deletion of HIF-1α in T Lymphocytes Results in Loss of Anti-tumor Activity in Models of Cancer Immunotherapy

To determine whether HIF-1α affects the acquisition of effector function in an antigen-specific model, we used transgenic OT-I expressing mice (Hogquist et al., 1994) with conditional HIF-1α mutations, and with or without dlck^{CRE} expression, to obtain $CD8^+$ T cells; these were then activated with cognate SIINFEKL peptide. Similar to the polyclonal αCD3/CD28 activated $CD8^+$ T cells, these transgenic T cells were able to proliferate and survive during the differentiation into effectors (Figure S3A), but had defects in CD62L downregulation (Figures 4A and S3B). HIF-1α mutant T cells also have altered granzyme B, TNFα, and IFNγ production after antigenic restimulation (Figures S3C and S3D), and expression of costimulatory/checkpoint receptors (Figures 4B and S3E), which was dependent on the level of oxygenation (Figure 4B). This is an important observation given the levels of oxygen present in lymphoid organs and solid tumors (Table S1). We checked the ability of HIF-1α-deficient OT-1 cells to migrate *in vivo* by cotransferring control $CD45.1^+$ and mutant $CD45.1^+$ $TdTomato^+$ $CD8^+$ T cells into CD45.2 hosts bearing B16-OVA tumors, then determining the relative percentage of each genotype of the transferred cells present in spleens, lymph nodes, and tumors 48 hr after adoptive transfer. HIF-1α-deficient T cells show both increased migration to lymphoid organs and a defect in homing to tumors (Figure 4C). When HIF-1α-deficient OT-I cells were transferred into B16-OVA tumor-bearing WT recipient mice, they were completely deficient in their ability to control tumor growth, leading to decreased survival (Figure 4D). Importantly, HIF-1α mutant mice harboring MC38 colon carcinoma tumors had a significantly inhibited response to αPD-1 and αCTLA4 combinatorial antibody-based immunotherapy relative to control WT littermates (Figure 4E).

CD8A mRNA in Human Breast Cancer Inversely Correlates with VEGF-A Expression

Based on our finding that loss of HIF-1α in T cells leads to decreased T cell infiltration and alterations in tumor vascularization, and given the high levels of VEGF-A produced by $CD8^+$ T cells, we investigated the correlations between the presence of total *VEGFA* transcripts and immunogenes in primary human breast cancer samples from a cohort of patients with breast cancer (the Uppsala cohort) (Miller et al., 2005). Figure 5A shows the correlation of transcription abundance values of *CD8A* versus *VEGFA*, carbonic anhydrase 9 (*CA9*), and endothelial metagene probes in the Uppsala cohort. The presence of *VEGFA* transcript in primary tumors negatively correlates with immunogenes associated with T cell infiltration. Among all tested pairwise comparisons between immunogenes and *VEGFA*, *CD8A* has the highest Spearman negative correlation. Figure 5B shows the correlation of *CD8A* versus *VEGFA* in different breast cancer subtypes. To further explore the clinical significance of these findings, we investigated the disease-free specific survival classified by breast cancer subtype according to the levels of *VEGFA* and *CD8A* transcript abundance in the Uppsala cohort (Figure S4). We then studied potential correlations between the levels of *CD8A* and a metagene comprising endothelial transcripts in breast cancer patients, before and after treatment with agents targeting the VEGF/VEGF receptor pathway: sunitinib (Figures 5C and 5D) and bevacizumab (PROMIX phase 2 trial) (Figures 5E and 5F). As can be seen, both treatments resulted in an increase in the expression of *CD8A*, which was correlated with an increase in the expression of the endothelial metagene.

VEGF-A Deficiency in T Lymphocytes Accelerates Isograft Tumor Growth

Given the potential correlation between T cell responses and tumor vascularization, we next determined how the HIF target VEGF-A might be affecting tumor progression through its expression in $CD8^+$ T cells. We deleted the *Vegfa* gene in T cells using the same transgenic driver of cre expression that is described above. This efficiently ablated the production of VEGF-A in these cells (Figures S5A–S5D); the later stage of lymphoid deletion in the dlck-cre prevented effects on T cell development, which were described in experiments where VEGF-A was deleted earlier in lymphoid differentiation (Gerber et al., 2002; Ohm et al., 2003) (Figures S5E–S5H).

To first determine whether VEGF-A affects T cell function more generally, we cultured CTLs obtained from VEGF$^{fl/fl}$dlck^{CRE} mice under 21% O_2 and 1% O_2. These mutant cells had no alteration of survival, glycolytic metabolism, or expression of cytolytic and costimulatory/checkpoint receptors, relative to those same parameters in WT cells. VEGF-A-deficient OT-1 CTLs exhibit similar CD62L and CD44 expression levels relative to those

Figure 4. HIF-1α Is Necessary for Effector $CD8^+$ T Cell Function and Migration

(A) $CD8^+$ T cells were isolated from spleens of OT-1 HIF-1α$^{fl/fl}$dlck^{CRE} (maroon) and littermate control (black) mice and activated with cognate peptide for 2 days, then expanded for 5 days in the presence of IL-2 and subjected to 21%, 5%, or 1% O_2 for 24 hr. Representative flow-cytometry histograms showing the expression of CD44 and CD62L (n = 3).

(B) Expression of intracellular granzyme B and the indicated costimulatory molecules/checkpoint receptors on $CD8^+$ T cells prepared as in (A).

(C) Left: diagram outlining the *in vivo* migration experiment: representative flow-cytometry plots are shown for each pool before and after adoptive cotransfer of HIF-1α mutant ($CD45.1^{+/}$$TdTomato^+$, maroon) and control ($CD45.1^+$, black) OT-1 T cells into $CD45.2^+$ B16-OVA tumor-bearing WT mice. Right: spleens, lymph nodes (LN), and tumors were collected 48 hr after the adoptive cell cotransfer and relative percentages of migrated cells of the indicated genotypes are shown (error bars represent SD, n = 6).

(D) 1×10^6 OT-1 cells were transferred into recipient mice harboring B16-OVA tumors (n = 6): tumor volumes (left) and overall percent survival (right) are shown; statistical analysis by log-rank (Mantel-Cox) test; error bars represent SEM.

(E) Tumor growth curves of MC38 tumor cells subcutaneously injected into HIF-1α mutant and littermate controls. Mice were treated with either isotype control antibodies (left, n = 3) or a combination of αPD-1 and αCTLA4 blocking antibodies (right, n = 6), on days 5, 7, and 9 (dashed lines). Statistical analysis was performed by two-way ANOVA with Sidak correction for multiple comparisons. Tumor volumes are shown (mean values ± SEM).

$^{****}p < 0.00005$, $^{**}p < 0.005$, $^{*}p < 0.05$; n.s., not significant. See also Figure S3.

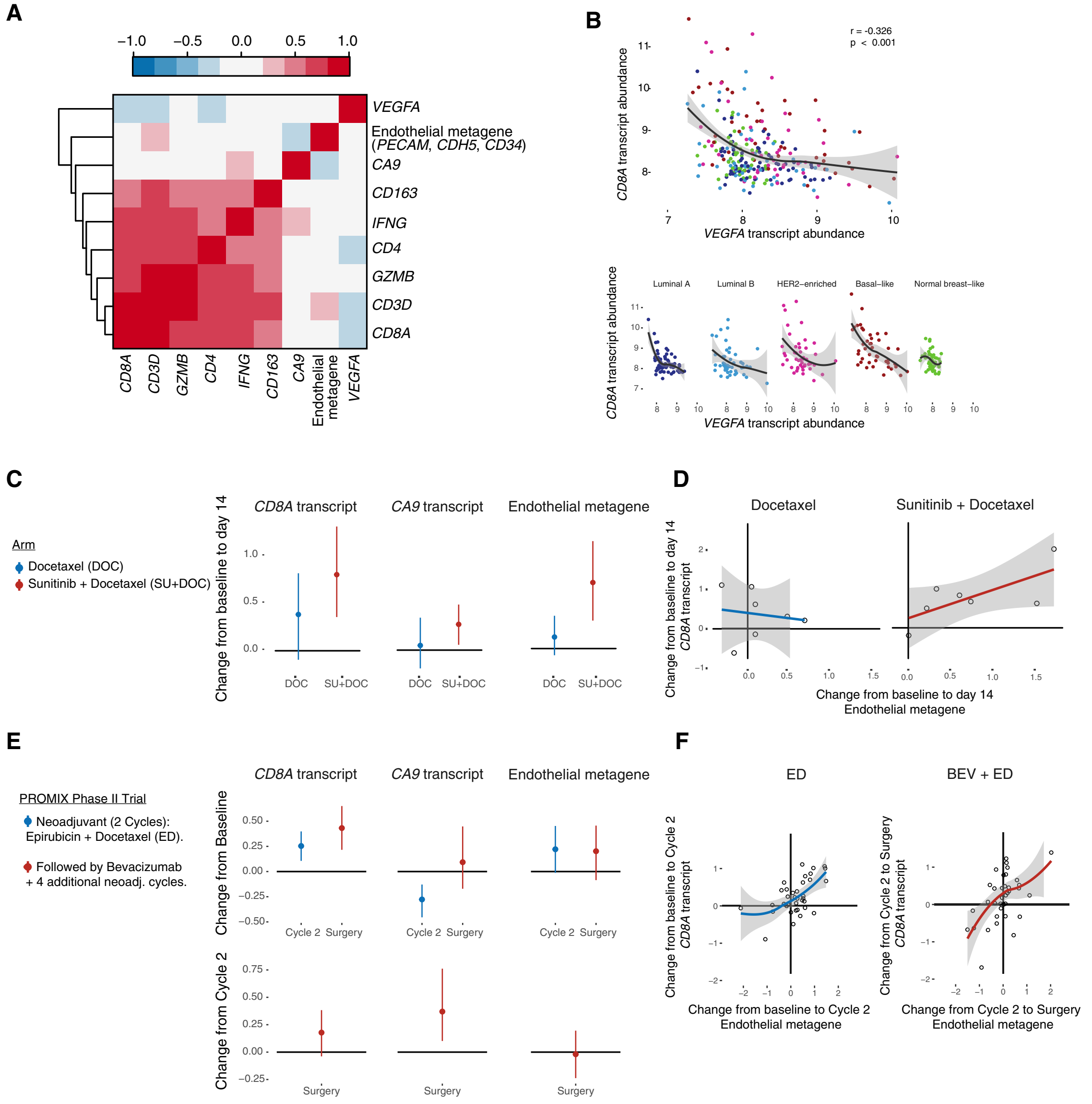

Figure 5. *CD8A* Transcript Abundance in Human Breast Cancer Positively Correlates with an Endothelial Cell Metagene

(A) Correlation analyses between *VEGFA*, *CA9*, and an endothelial metagene (*PECAM*, *CDH5*, *CD34*); and a set of immunogenes in the Uppsala cohort. Colorgram of Spearman's rank correlation matrix where the gene transcript levels are arranged according to hierarchical clustering. Red indicates positive correlation and blue, negative correlation. *CD8A* versus endothelial metagene Spearman's correlation coefficient rho = 0.191, p = 0.0023.

(B) Correlation between *CD8A* and *VEGFA* transcript abundance in the Uppsala cohort, and according to breast cancer subtype (mean values with confidence interval).

(C) Docetaxel- and sunitinib + docetaxel-induced changes from baseline in the expression of the indicated transcripts and metagene 14 days after treatment in breast cancer patients (vertical lines represent 95% confidence intervals).

(D) Correlations between the changes in *CD8A* transcript and endothelial metagene abundance from baseline to 14 days after the indicated treatments (mean values with confidence interval).

(E) Her2-negative breast cancer patients received two cycles of epirubicin and docetaxel (ED), followed by ED plus bevacizumab for four additional cycles (BEV + ED). Change in the expression of the indicated transcripts and metagene from baseline to the second cycle and to surgery (top), and from the second cycle to surgery (bottom). Multi-center, single arm, phase 2 PROMIX clinical trial; vertical lines represent 95% confidence intervals.

(F) Correlations between the changes in *CD8A* transcript and endothelial metagene abundance from baseline to second cycle (epirubicin + docetaxel) and second cycle to surgery (bevacizumab + epirubicin + docetaxel) in the PROMIX trial (mean values with confidence interval). Transcript abundance values represent log_2-transformed probe intensity values.

See also Figure S4.

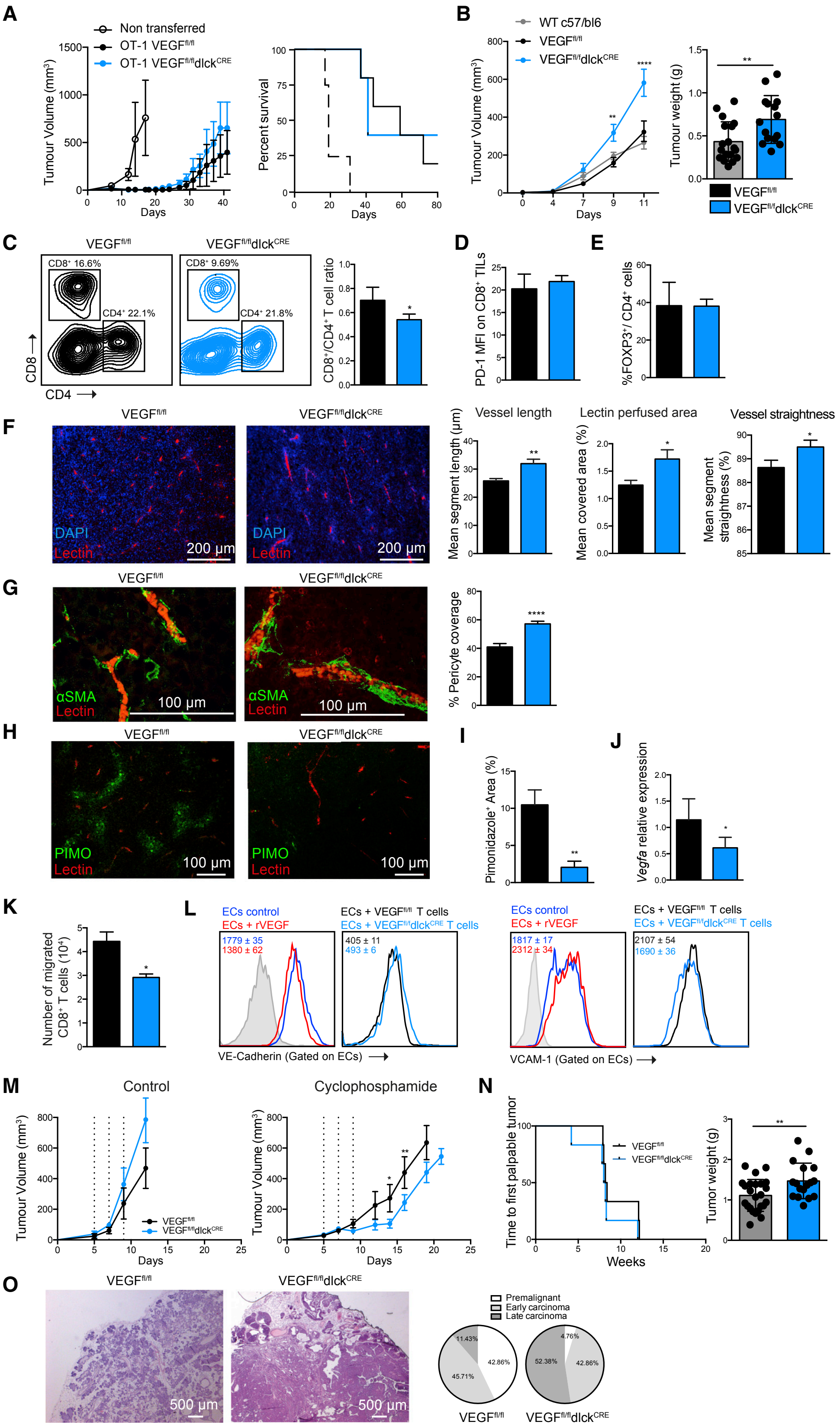

(legend on next page)

seen in WT T cells, and have only a minor reduction in expression of costimulatory molecules (Figures S6A–S6D). This reduction did not prevent the VEGF-A mutant cells from carrying out efficient killing of ovalbumin-expressing target cells in an *in vitro* assay (Figure S6E).

To determine whether directed killing of tumor cells by T cells was influenced by expression of VEGF-A, we adoptively transferred $VEGF^{fl/fl}dlck^{CRE}$ or WT CTLs to mice harboring B16-OVA tumors. VEGF-A deficiency only marginally affected tumor growth in this model (Figure 6A). Directed tumor cell killing by T cells is not affected by the loss of T cell VEGF-A, indicating that this aspect of HIF-1α function is not VEGF-A dependent.

Subcutaneous LLC tumors grew at a faster rate in $VEGF^{fl/fl}dlck^{CRE}$ mutant mice than that seen in WT control mice (Figure 6B), at rates comparable with the tumor growth rate in mice lacking VEGF-A in the myeloid compartment ($VEGF^{fl/fl}LysM^{CRE}$, Figures S6F and S6G). This latter observation was previously documented as being related to an increased degree of tumor perfusion (Stockmann et al., 2008), which led to significantly increased tumor weights in myeloid VEGF-A null animals at endpoint.

Analyses of the composition of polyclonal tumor-infiltrating lymphocytes lacking VEGF-A revealed a decreased rate of $CD8^+$ T cells migrating into LLC tumors (Figure 6C). In contrast to HIF-1α-deficient tumor-infiltrating $CD8^+$ T cells, however, PD-1 expression in VEGF-deficient tumor-infiltrating lymphocytes (TILs) is unaffected (Figure 6D) and there are no changes in FOXP3 expression levels in infiltrating $CD4^+$ T cells (Figure 6E).

Tumor vessels in mice lacking T cell-derived VEGF-A exhibited some of the hallmarks of normalization, including increased perfusion and segment length between branch points, and decreased tortuosity (Figure 6F), as well as increased pericyte coverage of tumor blood vessels (Figure 6G). Interestingly, the overall level of hypoxia was decreased, as shown by staining with piminidazole (Figures 6H, 6I, and S6H). Consistent with better perfusion, the overall expression of *Vegfa* mRNA in the total tumor lysate is also decreased in $VEGF^{fl/fl}dlck^{CRE}$ mutant mice (Figure 6J).

The lower levels of infiltrating lymphocytes in tumors in VEGF-A mutant animals suggests that VEGF-A secreted by $CD8^+$ T cells might affect T cell homing through endothelial barriers. An *in vitro* migration assay performed with T cells isolated from VEGF-A mutant mice shows impaired migration across a primary endothelial cell monolayer after culture in hypoxia, supporting this hypothesis (Figure 6K), and gave results similar to those seen in the same experimental coculture model described above employing HIF-1α-deficient $CD8^+$ T cells.

We then explored mechanisms involved in cell migration: T cell-derived VEGF-A induced VE-cadherin endocytosis in endothelial cells, and gave rise to a higher expression of the adhesion molecule VCAM-1 (Figure 6L), an observation consistent with the increased homing to tumors of VEGF-A-expressing T cells.

To determine whether the described vascular changes, consistent with vascular normalization, affect the delivery and efficacy of chemotherapies to the tumor, we administered cyclophosphamide to mutant and control tumor-bearing mice. Loss of T cell-derived VEGF-A increased the chemotherapeutic effect of the treatment (Figure 6M); as we did not assay for total amount of cyclophosphamide in the treated tumors, this could be the result of a change in delivery of the molecule or a change in a secondary mediator of cytotoxicity.

Figure 6. VEGF-A Deletion in T Lymphocytes Accelerates Tumor Growth and Normalizes Tumor Vasculature

(A) 1×10^6 OT-1 cells of the indicated genotype were adoptively transferred into WT recipient mice harboring B16-OVA tumors (n = 6). Left: tumor growth on recipient mice (error bars represent SE). Right: overall survival.

(B) Left: representative growth curve of subcutaneously injected LLC tumors in $VEGF^{fl/fl}dlck^{CRE}$ (blue), $VEGF^{fl/fl}$ littermate controls (gray), and WT c57/bl6 control mice (black) (0.5×10^6 tumor cells/mouse) (error bars represent SE). Right: tumor weights at the endpoint of the experiment (n > 13 per group, error bars represent SD).

(C) Representative dot plots showing $CD8^+$ and $CD4^+$ polyclonal T cell LLC tumor infiltration in mutant mice (blue) and littermate controls (black) by flow cytometry. Right: $CD8^+$ to $CD4^+$ ratio in TILs (n = 6, error bars represent SD).

(D) PD-1 expression on $CD8^+$ TILs (n = 6, error bars represent SD).

(E) Percent $FOXP3^+$ cells on $CD4^+$ TILs (n = 6, error bars represent SD).

(F) Left: immunofluorescence images of LLC tumor sections (blue = DAPI, red = tomato lectin-Dylight-594). Right: analyses of vessel length, percent perfused area, and vessel straightness of LLC tumors grafted in $VEGF^{fl/fl}dlck^{CRE}$ (blue) or control (black) mice injected with tomato lectin-Dylight-594. The means of these parameters per mouse are shown (4 fields per mouse, 1 field = 0.8 mm^2, n $\geq$ 6 mice per group, error bars represent SEM).

(G) Left: representative confocal images of LLC tumor sections for the indicated genotypes (red = tomato lectin-Dylight-594, green = α-SMA-AF488). Right: quantification of the pericyte coverage (4–6 fields per mouse, n $\geq$ 6 mice per group, error bars represent SEM).

(H) Representative confocal images of LLC tumor sections stained with pimonidazole (green).

(I) Quantification of percent $pimonidazole^+$ areas in LLC isografts (n $\geq$ 6 mice per group, error bars represent SEM).

(J) *Vegfa* relative expression in total LLC tumor lysates by qRT-PCR (error bars represent SD).

(K) *In vitro* T cell migration of VEGF-A-deficient (blue) and control (black) $CD8^+$ T cells through a confluent layer of primary endothelial cells (n = 4, error bars represent SD).

(L) Flow-cytometry histograms showing the surface VE-cadherin (left) and VCAM-1 (right) expression on MS-1 endothelial cells after 2 hr of treatment with recombinant VEGF-A (20 ng/mL, red) or control (dark blue); and surface expression of VE-cadherin and VCAM-1 after 2 hr of coculture with $CD8^+$ lymphocytes of the indicated genotypes. MFI and ±SD values are shown for each histogram.

(M) Growth curve of subcutaneous LLC tumors treated with three doses of vehicle control (n = 3, left) or cyclophosphamide (170 mg/kg, n = 7, right). Error bars represent SEM.

(N) Time to first palpable tumors for the indicated genotypes in a transgenic model of spontaneous mammary adenocarcinoma (MMTV-PYMT) (error bars represent SD) (left) and total tumor weights at 17 weeks of age (right, n $\geq$ 17 mice per group).

(O) H&E staining and percent PYMT-MMTV tumor progression stages for the indicated genotypes.

A two-tailed unpaired Student's t test was used for comparisons. For tumor volumes, statistical analysis was performed by two-way ANOVA with Sidak correction for multiple comparisons. ****$p < 0.00005$, **$p < 0.005$, *$p < 0.05$. See also Figures S5 and S6.

VEGF-A Deletion in T Lymphocytes Accelerates Tumorigenesis

To ascertain the role of T cell-derived VEGF-A in a spontaneous transgenic model of cancer, we employed the MMTV-PyMT model of multifocal mammary adenocarcinoma (Guy et al., 1992) backcrossed to a C57/Bl6 background (Davie et al., 2007). Tumor latency, or time to the first palpable tumor, is similar in VEGF-A mutant or WT littermate controls (Figure 6N). However, at 17 weeks of age, total tumor mass is significantly increased in mice lacking T cell-derived VEGF-A (Figure 6N), and tumorigenic progression is significantly advanced when scored histologically (Figure 6O).

DISCUSSION

There is a wealth of cell types that express HIF transcription factors in solid tumors. This is due to both the hypoxic nature of solid tumors themselves and to other mechanisms for inducing HIF expression, e.g., cytokine induction of *HIF1A* transcription. A key to a better understanding of and, ultimately, therapy for cancer is an increased characterization of the interactions between the varying responses of differing cell types in tumors to the physiological challenges of malignant growth.

We show here that $CD8^+$ T cells express both of the primary HIF-α transcription factors. We also show that following activation, the angiogenic factor VEGF-A is produced at high levels in $CD8^+$ T cells. Although HIF-1α expression in T cells is linked with a number of mechanisms for expression of T cell effector molecules, we have now demonstrated that at least one part of the role for HIF-1α in $CD8^+$ T cells is to regulate a VEGF-A response, one that in turn directly modulates tumorigenesis.

It has been shown that VHL deficiency in T cells promotes the acquisition of effector function, leading to enhanced viral clearance but also an exacerbated immune response (Doedens et al., 2013). Deficiency of the three oxygen-sensing prolyl-hydroxylase enzymes (PHD1, PHD2, and PHD3) in T cells also promotes T cell effector function (Clever et al., 2016). However, it is also clear that the loss of these negative regulators affects a range of downstream functions in cells and tissues.

The HIF-1α-driven transcriptional program that drives glycolytic metabolism under hypoxia is impaired in HIF-1α-deficient T cells, and it is clear that a high glycolytic rate is a prominent feature of effector $CD8^+$ T cells (Cham and Gajewski, 2005; Chang et al., 2013). Here, we show that HIF-1α is essential for controlling effector cell differentiation, migration, and function, as well as the expression of numerous costimulatory and exhaustion-related receptors in the tumor microenvironment. Specific HIF-1α deletion in T cells resulted in defective effector differentiation and accelerated tumor growth, once again demonstrating that the contribution of HIF to cancer progression is dependent on the cellular context.

We found that HIF-1α deletion resulted in impaired expression of a set of proteins critically involved in CTL-mediated tumor rejection. The production of effector cytokines, including IFNγ and TNFα and cytolytic molecules such as granzyme B, were reduced as a result of HIF-1α genetic ablation. These immunologic molecules are key in immunosurveillance (Kaplan et al., 1998; Shankaran et al., 2001). Costimulatory and checkpoint receptors, such as CD137, GITR, and OX40, and TIM3, PD-1, CTLA-4, and LAG-3 are another set of HIF-1α-dependent proteins expressed by CTLs and involved in the fine-tuning of the immune response. Due to the inhibitory role of checkpoint receptors on T cells, they are targets for immunotherapy (Sharma and Allison, 2015). Among them, we identified that PD-1 expression in tumor-infiltrating lymphocytes was HIF-1α dependent, an important observation given that PD-1 identifies the patient-specific $CD8^+$ tumor-reactive TIL repertoire (Gros et al., 2014). This differs from the data described in Doedens et al. (2013), where we saw that downregulation of PD-1 was VHL dependent but that this was not ablated in VHL/HIF-1α/HIF-2α triple-deficient cells; this result thus indicates that PD-1 downregulation is not due to the direct activity of either of the HIFα factors.

After activation upon antigen presentation in secondary lymphoid organs, T cells home to tumors. Different subsets of T cells found in tumors can correlate with differing clinical outcomes and prognosis (Galon et al., 2013). As a result of *Hif1a* deletion in T cells, the ratio of $CD8^+$ to $FOXP3^+$ cells in TILs was significantly reduced. We found that this is correlated with impaired $CD8^+$ T cell homing to tumors. This was consistent with our finding that $CD8^+$ T cells lacking HIF-1α exhibit reduced migration through endothelial barriers.

$FOXP3^+$ regulatory T regulatory (Treg) cells have an important role in immune homeostasis and cancer. The VHL-HIF axis is essential for Treg function, as HIF-1α promotes FOXP3 proteasomal degradation (Dang et al., 2011; Shi et al., 2011) and binds to the IFNγ promoter to drive T helper 1 responses (Lee et al., 2015). We show a decreased ratio of IFNγ^+ to $FOXP3^+$ cells in $CD4^+$ TILs lacking HIF-1α, which could further contribute to the observed accelerated tumor growth. This supports a model wherein HIF-1α increases overall effector T cell response. We show here also that HIF-1α can influence immunotherapy, an important observation given the recent success of autologous cell-based cancer immunotherapies (June et al., 2015).

Immunological and angiogenic pathways influence many aspects of cancer development, and their interrelationships are complex. We provide evidence that low levels of VEGF-A and high levels of CD8 in the tumor microenvironment are predictors of disease-free survival in breast cancer patients, corroborating previous studies (DeNardo et al., 2011; Linderholm et al., 2003). Our data show that the level of VEGF-A in tumors inversely correlates with the presence of $CD8^+$ T cells. As the primary source of VEGF-A in a tumor will often be the malignant cells themselves, the relationship between the tumor, infiltrating TILs, and the production of VEGF-A is difficult to place in an etiologic order given our observations.

However, we show that *Vegfa* deletion in $CD8^+$ T cells leads to enhanced tumor growth. This does not appear to be due to an intracrine effect of VEGF-A in $CD8^+$ T cells, and we rule out a cell-intrinsic defect of VEGF-deficient CTLs in the acquisition of an effector phenotype as a direct explanation for the enhanced tumor growth. VEGF-A production in tumors has been linked to $CD4^+$ $FOXP3^+$ Treg cell abundance (Hansen et al., 2012; Terme et al., 2013) and decreased infiltration by tumor-reactive $CD8^+$ T cells (Peng et al., 2016). VEGF-A production by $FOXP3^+$ Treg cells also contributes to tumor angiogenesis *in vivo* (Facciabene et al., 2011).

We found that VEGF-A-deficient $CD8^+$ T cells do not infiltrate tumors efficiently. VEGF-A is known to be an important

regulator of tumor/endothelial cell adhesion properties, and plays a crucial role in the interactions between immune and vascular cells, acting to promote recruitment (Detmar et al., 1998; Kim et al., 2001; Melder et al., 1996). Systemic chronic blockade of VEGF-A has been shown to increase T cell homing to tumors (Motz et al., 2014). Our data would indicate that these previously documented effects are not related to the expression of VEGF-A in T cells themselves, but are likely part of a signaling/homing mechanism in the tumor cells or in other stromal cells.

We show that loss of T cell-derived VEGF-A gives rise to increased tumor oxygenation/perfusion, increased pericyte coverage of tumor blood vessels, and accelerated tumor growth. Vascular normalization is usually associated with decreased hypoxia, better perfusion of the tumor, and a shift in cancer cell metabolism, although it is clear that vascular normalization does not always lead to increased tumor growth and can also result in reduced or unaffected growth (Carmeliet and Jain, 2011; Claesson-Welsh and Welsh, 2013). Tumor response to chemotherapy is known to be affected by the levels of oxygenation and the ability of the vessel network to deliver drugs (Goel et al., 2012). In this context, we show that mice lacking T cell-derived VEGF-A are more sensitive to chemotherapy. As a result of better perfusion, hypoxia and, thus, VEGF-A expression by other cells infiltrating the tumor would also be reduced. Among them, macrophages actively contribute to the angiogenic switch (Lin et al., 2006) by secreting VEGF-A (Stockmann et al., 2008). Lymphocyte-derived VEGF-A effects on macrophages and lymphocyte-macrophage interactions could further explain the observed vascular phenotype (Ramello et al., 2014), and this will be an important subject for future investigation.

Our findings provide further rationale for the ongoing clinical evaluation of combinatorial therapies comprising immunotherapies and anti-angiogenic approaches (Hodi et al., 2014; Wu et al., 2016). In summary, we show by T cell-specific gene deletion that HIF-1α is an essential regulator of T cell effector responses in the tumor microenvironment. Furthermore, we show that this occurs in part, but not solely, through HIF-1α regulation of VEGF-A; and that VEGF-A in effector CD8$^+$ T cells contributes to T cell infiltration, tumor vascularization, and tumor progression.

STAR★METHODS

Detailed methods are provided in the online version of this paper and include the following:

- KEY RESOURCES TABLE
- CONTACT FOR REAGENT AND RESOURCE SHARING
- EXPERIMENTAL MODELS AND SUBJECT DETAILS
  - Animal Models
  - Cell Culture and Cellular Assays
  - Genomic Analyses of Breast Cancer Samples
- METHODS DETAILS
  - Metabolism Assays
  - Cytotoxicity Assays
  - Transendothelial T-Cell Migration Assays
  - Surface VCAM-1 and VE-Cadherin Analysis
  - Flow Cytometry
  - QRT-PCR and Western Blotting
  - Tumor Growth
  - Adoptive Cell Transfer
  - *In Vivo* Immunotherapy and Chemotherapy
  - Histology, Immunofluorescence and *In Vivo* pO_2 Measurements
- STATISTICAL ANALYSIS
  - Statistical Analysis
  - Clinical Gene-Expression Data Analysis

SUPPLEMENTAL INFORMATION

Supplemental Information includes six figures and three tables and can be found with this article online at https://doi.org/10.1016/j.ccell.2017.10.003.

AUTHOR CONTRIBUTIONS

A.P. designed and performed experiments, analyzed the data, and wrote the manuscript. P.A.T. performed experiments and analyzed the data. H.R., S.F., N.V., D.M., and P.V. designed and performed experiments and contributed ideas. A.T.P. and A.W.G. analyzed data and contributed ideas. J.L., T.F., N.L., I.H., T.H., and J.B. designed experiments, analyzed data, contributed ideas, and wrote the manuscript. R.S.J. designed experiments, analyzed data, wrote the manuscript, and administered the project.

ACKNOWLEDGMENTS

Funding has been provided by a Wellcome Trust Principal Research Fellowship (WT092738MA), the Swedish Cancer Fund (Cancerfonden), the Swedish Childrens' Cancer Fund (Barncancerfonden), and the Swedish Medical Research Council (Vetenskapsrådet) for R.S.J., the US National Institutes of Health for A.W.G. (A1096852, A1072117), and a Marie Curie IEF fellowship for A.P. Support is acknowledged from Lund University BioCARE for N.L and I.H. We thank Cristina Branco, Jingwei Sim, Colin Evans, and Renato Colaço for discussions and experimental assistance.

Received: September 3, 2016
Revised: May 18, 2017
Accepted: October 4, 2017
Published: November 13, 2017; corrected online November 15, 2017

REFERENCES

Bergh, J., Bondarenko, I.M., Lichinitser, M.R., Liljegren, A., Greil, R., Voytko, N.L., Makhson, A.N., Cortes, J., Lortholary, A., Bischoff, J., et al. (2012). First-line treatment of advanced breast cancer with sunitinib in combination with docetaxel versus docetaxel alone: results of a prospective, randomized phase III study. J. Clin. Oncol. *30*, 921–929.

Branco-Price, C., Zhang, N., Schnelle, M., Evans, C., Katschinski, D.M., Liao, D., Ellies, L., and Johnson, R.S. (2012). Endothelial cell HIF-1alpha and HIF-2alpha differentially regulate metastatic success. Cancer Cell *21*, 52–65.

Buck, M.D., O'Sullivan, D., and Pearce, E.L. (2015). T cell metabolism drives immunity. J. Exp. Med. *212*, 1345–1360.

Carmeliet, P., and Jain, R.K. (2011). Principles and mechanisms of vessel normalization for cancer and other angiogenic diseases. Nat. Rev. Drug Discov. *10*, 417–427.

Cham, C.M., and Gajewski, T.F. (2005). Metabolic mechanisms of tumor resistance to T cell effector function. Immunol. Res. *31*, 107–118.

Chang, C.H., Curtis, J.D., Maggi, L.B., Jr., Faubert, B., Villarino, A.V., O'Sullivan, D., Huang, S.C., van der Windt, G.J., Blagih, J., Qiu, J., et al. (2013). Posttranscriptional control of T cell effector function by aerobic glycolysis. Cell *153*, 1239–1251.

Claesson-Welsh, L., and Welsh, M. (2013). VEGFA and tumour angiogenesis. J. Intern. Med. *273*, 114–127.

Clever, D., Roychoudhuri, R., Constantinides, M.G., Askenase, M.H., Sukumar, M., Klebanoff, C.A., Eil, R.L., Hickman, H.D., Yu, Z., Pan, J.H., et al. (2016). Oxygen sensing by T cells establishes an immunologically tolerant metastatic niche. Cell *166*, 1117–1131.e14.

Cramer, T., Yamanishi, Y., Clausen, B.E., Forster, I., Pawlinski, R., Mackman, N., Haase, V.H., Jaenisch, R., Corr, M., Nizet, V., et al. (2003). HIF-1alpha is essential for myeloid cell-mediated inflammation. Cell *112*, 645–657.

Dang, E.V., Barbi, J., Yang, H.Y., Jinasena, D., Yu, H., Zheng, Y., Bordman, Z., Fu, J., Kim, Y., Yen, H.R., et al. (2011). Control of T(H)17/T(reg) balance by hypoxia-inducible factor 1. Cell *146*, 772–784.

Davie, S.A., Maglione, J.E., Manner, C.K., Young, D., Cardiff, R.D., MacLeod, C.L., and Ellies, L.G. (2007). Effects of FVB/NJ and C57Bl/6J strain backgrounds on mammary tumor phenotype in inducible nitric oxide synthase deficient mice. Transgenic Res. *16*, 193–201.

DeNardo, D.G., Brennan, D.J., Rexhepaj, E., Ruffell, B., Shiao, S.L., Madden, S.F., Gallagher, W.M., Wadhwani, N., Keil, S.D., Junaid, S.A., et al. (2011). Leukocyte complexity predicts breast cancer survival and functionally regulates response to chemotherapy. Cancer Discov. *1*, 54–67.

Detmar, M., Brown, L.F., Schon, M.P., Elicker, B.M., Velasco, P., Richard, L., Fukumura, D., Monsky, W., Claffey, K.P., and Jain, R.K. (1998). Increased microvascular density and enhanced leukocyte rolling and adhesion in the skin of VEGF transgenic mice. J. Invest. Dermatol. *111*, 1–6.

Doedens, A.L., Phan, A.T., Stradner, M.H., Fujimoto, J.K., Nguyen, J.V., Yang, E., Johnson, R.S., and Goldrath, A.W. (2013). Hypoxia-inducible factors enhance the effector responses of CD8(+) T cells to persistent antigen. Nat. Immunol. *14*, 1173–1182.

Facciabene, A., Peng, X., Hagemann, I.S., Balint, K., Barchetti, A., Wang, L.P., Gimotty, P.A., Gilks, C.B., Lal, P., Zhang, L., and Coukos, G. (2011). Tumour hypoxia promotes tolerance and angiogenesis via CCL28 and T(reg) cells. Nature *475*, 226–230.

Finlay, D.K., Rosenzweig, E., Sinclair, L.V., Feijoo-Carnero, C., Hukelmann, J.L., Rolf, J., Panteleyev, A.A., Okkenhaug, K., and Cantrell, D.A. (2012). PDK1 regulation of mTOR and hypoxia-inducible factor 1 integrate metabolism and migration of $CD8^+$ T cells. J. Exp. Med. *209*, 2441–2453.

Foukakis, T., Lovrot, J., Sandqvist, P., Xie, H., Lindstrom, L.S., Giorgetti, C., Jacobsson, H., Hedayati, E., and Bergh, J. (2015). Gene expression profiling of sequential metastatic biopsies for biomarker discovery in breast cancer. Mol. Oncol. *9*, 1384–1391.

Freeman, M.R., Schneck, F.X., Gagnon, M.L., Corless, C., Soker, S., Niknejad, K., Peoples, G.E., and Klagsbrun, M. (1995). Peripheral blood T lymphocytes and lymphocytes infiltrating human cancers express vascular endothelial growth factor: a potential role for T cells in angiogenesis. Cancer Res. *55*, 4140–4145.

Galon, J., Angell, H.K., Bedognetti, D., and Marincola, F.M. (2013). The continuum of cancer immunosurveillance: prognostic, predictive, and mechanistic signatures. Immunity *39*, 11–26.

Gerber, H.P., Malik, A.K., Solar, G.P., Sherman, D., Liang, X.H., Meng, G., Hong, K., Marsters, J.C., and Ferrara, N. (2002). VEGF regulates haematopoietic stem cell survival by an internal autocrine loop mechanism. Nature *417*, 954–958.

Goel, S., Wong, A.H., and Jain, R.K. (2012). Vascular normalization as a therapeutic strategy for malignant and nonmalignant disease. Cold Spring Harb. Perspect. Med. *2*, a006486.

Gros, A., Robbins, P.F., Yao, X., Li, Y.F., Turcotte, S., Tran, E., Wunderlich, J.R., Mixon, A., Farid, S., Dudley, M.E., et al. (2014). PD-1 identifies the patient-specific CD8(+) tumor-reactive repertoire infiltrating human tumors. J. Clin. Invest. *124*, 2246–2259.

Gruber, M., Hu, C.J., Johnson, R.S., Brown, E.J., Keith, B., and Simon, M.C. (2007). Acute postnatal ablation of Hif-2alpha results in anemia. Proc. Natl. Acad. Sci. USA *104*, 2301–2306.

Guy, C.T., Cardiff, R.D., and Muller, W.J. (1992). Induction of mammary tumors by expression of polyomavirus middle T oncogene: a transgenic mouse model for metastatic disease. Mol. Cell. Biol. *12*, 954–961.

Hansen, W., Hutzler, M., Abel, S., Alter, C., Stockmann, C., Kliche, S., Albert, J., Sparwasser, T., Sakaguchi, S., Westendorf, A.M., et al. (2012). Neuropilin 1 deficiency on CD4+Foxp3+ regulatory T cells impairs mouse melanoma growth. J. Exp. Med. *209*, 2001–2016.

Hodi, F.S., Lawrence, D., Lezcano, C., Wu, X., Zhou, J., Sasada, T., Zeng, W., Giobbie-Hurder, A., Atkins, M.B., Ibrahim, N., et al. (2014). Bevacizumab plus ipilimumab in patients with metastatic melanoma. Cancer Immunol. Res. *2*, 632–642.

Hogquist, K.A., Jameson, S.C., Heath, W.R., Howard, J.L., Bevan, M.J., and Carbone, F.R. (1994). T cell receptor antagonist peptides induce positive selection. Cell *76*, 17–27.

Jantsch, J., Chakravortty, D., Turza, N., Prechtel, A.T., Buchholz, B., Gerlach, R.G., Volke, M., Glasner, J., Warnecke, C., Wiesener, M.S., et al. (2008). Hypoxia and hypoxia-inducible factor-1 alpha modulate lipopolysaccharide-induced dendritic cell activation and function. J. Immunol. *180*, 4697–4705.

June, C.H., Riddell, S.R., and Schumacher, T.N. (2015). Adoptive cellular therapy: a race to the finish line. Sci. Transl. Med. *7*, 280ps287.

Kaplan, D.H., Shankaran, V., Dighe, A.S., Stockert, E., Aguet, M., Old, L.J., and Schreiber, R.D. (1998). Demonstration of an interferon gamma-dependent tumor surveillance system in immunocompetent mice. Proc. Natl. Acad. Sci. USA *95*, 7556–7561.

Kim, I., Moon, S.O., Park, S.K., Chae, S.W., and Koh, G.Y. (2001). Angiopoietin-1 reduces VEGF-stimulated leukocyte adhesion to endothelial cells by reducing ICAM-1, VCAM-1, and E-selectin expression. Circ. Res. *89*, 477–479.

Kim, J.W., Evans, C., Weidemann, A., Takeda, N., Lee, Y.S., Stockmann, C., Branco-Price, C., Brandberg, F., Leone, G., Ostrowski, M.C., and Johnson, R.S. (2012). Loss of fibroblast HIF-1alpha accelerates tumorigenesis. Cancer Res. *72*, 3187–3195.

Kim, J.W., Tchernyshyov, I., Semenza, G.L., and Dang, C.V. (2006). HIF-1-mediated expression of pyruvate dehydrogenase kinase: a metabolic switch required for cellular adaptation to hypoxia. Cell Metab. *3*, 177–185.

Lee, J.H., Elly, C., Park, Y., and Liu, Y.C. (2015). E3 Ubiquitin ligase VHL regulates hypoxia-inducible factor-1alpha to maintain regulatory T cell stability and suppressive capacity. Immunity *42*, 1062–1074.

Lin, E.Y., Jones, J.G., Li, P., Zhu, L., Whitney, K.D., Muller, W.J., and Pollard, J.W. (2003). Progression to malignancy in the polyoma middle T oncoprotein mouse breast cancer model provides a reliable model for human diseases. Am. J. Pathol. *163*, 2113–2126.

Lin, E.Y., Li, J.F., Gnatovskiy, L., Deng, Y., Zhu, L., Grzesik, D.A., Qian, H., Xue, X.N., and Pollard, J.W. (2006). Macrophages regulate the angiogenic switch in a mouse model of breast cancer. Cancer Res. *66*, 11238–11246.

Linderholm, B.K., Lindh, B., Beckman, L., Erlanson, M., Edin, K., Travelin, B., Bergh, J., Grankvist, K., and Henriksson, R. (2003). Prognostic correlation of basic fibroblast growth factor and vascular endothelial growth factor in 1307 primary breast cancers. Clin. Breast Cancer *4*, 340–347.

McNamee, E.N., Korns Johnson, D., Homann, D., and Clambey, E.T. (2013). Hypoxia and hypoxia-inducible factors as regulators of T cell development, differentiation, and function. Immunol. Res. *55*, 58–70.

Melder, R.J., Koenig, G.C., Witwer, B.P., Safabakhsh, N., Munn, L.L., and Jain, R.K. (1996). During angiogenesis, vascular endothelial growth factor and basic fibroblast growth factor regulate natural killer cell adhesion to tumor endothelium. Nat. Med. *2*, 992–997.

Miller, L.D., Smeds, J., George, J., Vega, V.B., Vergara, L., Ploner, A., Pawitan, Y., Hall, P., Klaar, S., Liu, E.T., and Bergh, J. (2005). An expression signature for p53 status in human breast cancer predicts mutation status, transcriptional effects, and patient survival. Proc. Natl. Acad. Sci. USA *102*, 13550–13555.

Motz, G.T., Santoro, S.P., Wang, L.P., Garrabrant, T., Lastra, R.R., Hagemann, I.S., Lal, P., Feldman, M.D., Benencia, F., and Coukos, G. (2014). Tumor endothelium FasL establishes a selective immune barrier promoting tolerance in tumors. Nat. Med. *20*, 607–615.

Nakamura, H., Makino, Y., Okamoto, K., Poellinger, L., Ohnuma, K., Morimoto, C., and Tanaka, H. (2005). TCR engagement increases hypoxia-inducible

factor-1 alpha protein synthesis via rapamycin-sensitive pathway under hypoxic conditions in human peripheral T cells. J. Immunol. *174*, 7592–7599.

Ohm, J.E., Gabrilovich, D.I., Sempowski, G.D., Kisseleva, E., Parman, K.S., Nadaf, S., and Carbone, D.P. (2003). VEGF inhibits T-cell development and may contribute to tumor-induced immune suppression. Blood *101*, 4878–4886.

Palazon, A., Goldrath, A.W., Nizet, V., and Johnson, R.S. (2014). HIF transcription factors, inflammation, and immunity. Immunity *41*, 518–528.

Papandreou, I., Cairns, R.A., Fontana, L., Lim, A.L., and Denko, N.C. (2006). HIF-1 mediates adaptation to hypoxia by actively downregulating mitochondrial oxygen consumption. Cell Metab. *3*, 187–197.

Pearce, E.L., Poffenberger, M.C., Chang, C.H., and Jones, R.G. (2013). Fueling immunity: insights into metabolism and lymphocyte function. Science *342*, 1242454.

Peng, W., Chen, J.Q., Liu, C., Malu, S., Creasy, C., Tetzlaff, M.T., Xu, C., McKenzie, J.A., Zhang, C., Liang, X., et al. (2016). Loss of PTEN promotes resistance to T cell-mediated immunotherapy. Cancer Discov. *6*, 202–216.

Ramello, M.C., Tosello Boari, J., Canale, F.P., Mena, H.A., Negrotto, S., Gastman, B., Gruppi, A., Acosta Rodriguez, E.V., and Montes, C.L. (2014). Tumor-induced senescent T cells promote the secretion of pro-inflammatory cytokines and angiogenic factors by human monocytes/macrophages through a mechanism that involves Tim-3 and CD40L. Cell Death Dis. *5*, e1507.

Ryan, H.E., Poloni, M., McNulty, W., Elson, D., Gassmann, M., Arbeit, J.M., and Johnson, R.S. (2000). Hypoxia-inducible factor-1alpha is a positive factor in solid tumor growth. Cancer Res. *60*, 4010–4015.

Seagroves, T.N., Ryan, H.E., Lu, H., Wouters, B.G., Knapp, M., Thibault, P., Laderoute, K., and Johnson, R.S. (2001). Transcription factor HIF-1 is a necessary mediator of the pasteur effect in mammalian cells. Mol. Cell. Biol. *21*, 3436–3444.

Semenza, G.L. (2012). Hypoxia-inducible factors in physiology and medicine. Cell *148*, 399–408.

Shankaran, V., Ikeda, H., Bruce, A.T., White, J.M., Swanson, P.E., Old, L.J., and Schreiber, R.D. (2001). IFNgamma and lymphocytes prevent primary tumour development and shape tumour immunogenicity. Nature *410*, 1107–1111.

Sharma, P., and Allison, J.P. (2015). The future of immune checkpoint therapy. Science *348*, 56–61.

Shi, L.Z., Wang, R., Huang, G., Vogel, P., Neale, G., Green, D.R., and Chi, H. (2011). HIF1alpha-dependent glycolytic pathway orchestrates a metabolic checkpoint for the differentiation of TH17 and Treg cells. J. Exp. Med. *208*, 1367–1376.

Shojaei, F., Zhong, C., Wu, X., Yu, L., and Ferrara, N. (2008). Role of myeloid cells in tumor angiogenesis and growth. Trends Cell Biol. *18*, 372–378.

Stockmann, C., Doedens, A., Weidemann, A., Zhang, N., Takeda, N., Greenberg, J.I., Cheresh, D.A., and Johnson, R.S. (2008). Deletion of vascular endothelial growth factor in myeloid cells accelerates tumorigenesis. Nature *456*, 814–818.

Takeda, N., O'Dea, E.L., Doedens, A., Kim, J.W., Weidemann, A., Stockmann, C., Asagiri, M., Simon, M.C., Hoffmann, A., and Johnson, R.S. (2010). Differential activation and antagonistic function of HIF-{alpha} isoforms in macrophages are essential for NO homeostasis. Genes Dev. *24*, 491–501.

Terme, M., Pernot, S., Marcheteau, E., Sandoval, F., Benhamouda, N., Colussi, O., Dubreuil, O., Carpentier, A.F., Tartour, E., and Taieb, J. (2013). VEGFA-VEGFR pathway blockade inhibits tumor-induced regulatory T-cell proliferation in colorectal cancer. Cancer Res. *73*, 539–549.

Vaupel, P., Kallinowski, F., and Okunieff, P. (1989). Blood flow, oxygen and nutrient supply, and metabolic microenvironment of human tumors: a review. Cancer Res. *49*, 6449–6465.

Walmsley, S.R., Print, C., Farahi, N., Peyssonnaux, C., Johnson, R.S., Cramer, T., Sobolewski, A., Condliffe, A.M., Cowburn, A.S., Johnson, N., and Chilvers, E.R. (2005). Hypoxia-induced neutrophil survival is mediated by HIF-1alpha-dependent NF-kappaB activity. J. Exp. Med. *201*, 105–115.

Wu, X., Giobbie-Hurder, A., Liao, X., Lawrence, D., McDermott, D., Zhou, J., Rodig, S., and Hodi, F.S. (2016). VEGF neutralization plus CTLA-4 blockade alters soluble and cellular factors associated with enhancing lymphocyte infiltration and humoral recognition in melanoma. Cancer Immunol. Res. *4*, 858–868.

Zhang, D.J., Wang, Q., Wei, J., Baimukanova, G., Buchholz, F., Stewart, A.F., Mao, X., and Killeen, N. (2005). Selective expression of the Cre recombinase in late-stage thymocytes using the distal promoter of the Lck gene. J. Immunol. *174*, 6725–6731.

Zhang, N., and Bevan, M.J. (2010). Dicer controls CD8+ T-cell activation, migration, and survival. Proc. Natl. Acad. Sci. USA *107*, 21629–21634.

STAR★METHODS

KEY RESOURCES TABLE

REAGENT or RESOURCE	SOURCE	IDENTIFIER
Antibodies		
Anti-mouse CD62L (clone MEL-14)	Biolegend	Cat# 104428
Anti-mouse CD44 (clone IM-7)	Biolegend	Cat# 103032
Anti-mouse CD8 (clone 53-6.7)	Biolegend	Cat# 100723
Anti-mouse CD3 (clone 17A2)	Biolegend	Cat# 100237
Anti-mouse CD4 (clone GK1.5)	Biolegend	Cat# 100408
Anti-mouse CD137 (clone 17B5)	Biolegend	Cat# 106110
Anti-mouse CD45.1 (clone A20)	Biolegend	Cat# 110728
Anti-mouse CD45.2 (clone 104)	Biolegend	Cat# 109814
Anti-mouse PD-1 (clone 29F.1A12)	Biolegend	Cat# 135214
Anti-mouse OX40 (clone OX-86)	Biolegend	Cat# 119414
Anti-mouse TIM-3 (clone B8.2C12)	Biolegend	Cat# 134006
Anti-mouse LAG-3 (clone C9B7W)	Biolegend	Cat# 125204
Anti-mouse GITR (clone DTA-1)	Biolegend	Cat# 126310
Anti-mouse Granzyme B (clone GB11)	Biolegend	Cat# 515406
Anti-mouse TNFα (clone MP6-XT22)	Biolegend	Cat# 506313
Anti-mouse IFNγ (clone XMG1.2)	Biolegend	Cat# 505808
Anti-mouse CD25 (clone 3C7)	Biolegend	Cat# 101910
Anti-mouse CD69 (clone H1.2F3)	Biolegend	Cat# 104507
Anti-mouse VCAM-1 (clone MVCAM.A)	Biolegend	Cat# 105718
Anti-mouse VE-Cadherin (clone BV13)	Biolegend	Cat# 138010
Anti-mouse/Rat FOXP3 (clone FJK-16 s)	Ebioscience	Cat# 17-5773
Anti-mouse F4/80 Microbeads	Miltenyi	Cat# 130-110-443
Anti-mouse CD8 Microbeads	Miltenyi	Cat# 130-049-401
Anti-mouse CD4 Microbeads	Miltenyi	Cat# 130-049-201
Anti-mouse rabbit polyclonal HIF-1α	Novus	Cat# NB100-449
Anti-mouse rabbit polyclonal HIF-2α	Novus	Cat# NB100-122
Anti-mouse rabbit polyclonal HDAC1	Abcam	Cat# Ab19845
Anti-mouse rabbit polyclonal β-actin	Abcam	Cat# ab8227
Anti-rabbit IgG HRP secondary antibody	RnD	Cat# HAF008
Anti-mouse CTLA-4 (clone 9H10)	BioXCell	Cat# BE0131
Anti-mouse PD-1 (clone RMP1-14)	BioXCell	Cat# BE0146
Polyclonal Syrian hamster IgG	BioXCell	Cat# BE0087
Rat IgG2a isotype control	BioXCell	Cat# BE0089
Anti-mouse SMA (clone 1A4)	Sigma	Cat# A5228
Anti-mouse IgG AF488 secondary antibody	ThermoFisher	Cat# R37120
LEAF purified anti-mouse CD3	Biolegend	Cat# 100331
LEAF purified anti-mouse CD28	Biolegend	Cat# 102102
Chemicals, Peptides, and Recombinant Proteins		
Ovalbumin (257-264) chicken	Sigma	Cat# S7951
Recombinant mouse IL-2	Biolegend	Cat# 575406
Recombinant mouse VEGF-A	RnD	Cat# 493-MV
Golgi stop protein transport inhibitor	BD	Cat# 554724
Cell stimulation cocktail	eBioscience	Cat# 00-4970-03
Cyclophosphamide	Sigma	Cat# C7397

(*Continued on next page*)

Continued		
REAGENT or RESOURCE	SOURCE	IDENTIFIER
Histoclear II	National diagnostics	Cat# HS-202
G418 Geneticin	ThermoFisher	Cat# 10131027
2-Mercaptoethanol	ThermoFisher	Cat# 21985023
Critical Commercial Assays		
RNeasy mini kit	QIAGEN	Cat# 74106
DNeasy kit	QIAGEN	Cat# 69506
NE-PER Nuclear and Cytoplasmic Extraction Reagents	Thermo Scientific	Cat# 78833
Seahorse Bioassay	Agilent	Cat# 102340
Seahorse cell energy phenotype kit	Agilent	Cat# 103325
Superscript III First strand synthesis kit	ThermoFisher	Cat# 18080051
DC Protein Assay	BioRad	Cat# 5000111
ECL prime western blotting	GE Healthcare	Cat# RPN2232
U-PLEX mouse VEGF-A Assay	MSD	Cat# K152-UVK
FOXP3 staining buffer set	eBioscience	Cat# 00-5523-00
Intracellular Fixation and Permeabilization buffer set	ThermoFisher	Cat# 88-8824-00
Absolute counting beads	ThermoFisher	Cat# C36950
Hypoxyprobe kit	Hpi Hypoxyprobe	Cat# HP6
DyLight 594 labeled Lycopersicon Esculentum lectin	Vector laboratories	Cat# DL-1177
Prolong Gold with DAPI	ThermoFisher	Cat# P36941
CFSE cell proliferation kit	ThermoFisher	Cat# C34554
LIVE/DEAD fixable violet kit	ThermoFisher	Cat# L34955
Experimental Models: Cell Lines		
MS-1	ATCC	Cat# CRL-2279
B16-F10	ATCC	Cat# CRL-6475
LLC	ATCC	Cat# CRL-1642
EG7-OVA	ATCC	Cat# CRL-2113
Mouse primary T lymphocytes	N/A	N/A
Experimental models: Organisms/Strains		
C57BL/6J	The Jackson Laboratory	000664
$Hif1a^{tm3Rsjo}$	In-house	N/A
$Epas1^{tm1Mcs}$	In-house	N/A
$Vegfa^{tm2Gne}$	In-house	N/A
Tg(Lck-icre)3779Nik	The Jackson Laboratory	012837
Tg(MMTV-PyVT)634Mul	The Jackson Laboratory	002374
$Gt(ROSA)26Sor^{tm9(CAG-tdTomato)Hze}$	The Jackson Laboratory	007909
$Ptprc^{a}Pepc^{b}$/BoyJ	The Jackson Laboratory	002014
Tg(TcraTcrb)1100Mjb	The Jackson Laboratory	003831
Oligonucleotides		
Primers (RT-PCR), see Table S2	Sigma-Aldrich	Custom
Primers (Deletion efficiency), see Table S3	IDT	Custom
Deposited Data		
Uppsala breast cancer cohort	NCBI Gene Expression Omnibus	GSE3494
Phase III Docetaxel+Sunitinib trial	NCBI Gene Expression Omnibus	GSE54323
Phase II PROMIX Bevacizumab trial	NCBI Gene Expression Omnibus	GSE87455

CONTACT FOR REAGENT AND RESOURCE SHARING

Further information and requests for resources and reagents should be directed to and will be fulfilled by the Lead Contact: Randall S. Johnson (rsj33@cam.ac.uk).

EXPERIMENTAL MODELS AND SUBJECT DETAILS

Animal Models

Mice carrying Hif1a, Epas1 and VEGF-A loxP-flanked alleles were crossed with dlck-cre or LysM-cre mice to obtain T cell specific gene deletion. Mice were backcrossed over ten generations to the C57bl/6 background. OT-1 mice containing transgenic inserts for mouse TCR-Vα2 and TCR-Vβ5 genes that recognise ovalbumin residues 257-264 (SIINFEKL) were crossed with CD45.1, Rosa-TdTomato, HIF-1$\alpha^{fl/fl}$dlck^{CRE}, VEGF$^{fl/fl}$dlck^{CRE} mice or their combinations. VEGF$^{fl/fl}$dlck^{CRE} mice were also crossed with transgenic mice expressing the polyoma middle T (PyMT) oncoprotein under the promoter of the mouse mammary tumor virus (MMTV) long terminal repeat (c57bl/6). Only female mice hemizygous for the PYMT oncogene were included in the tumor experiments. In all experiments, male and female mice between 6 and 20 weeks were used. Littermates negative for the cre recombinase expression were used as wild type controls. All experiments were carried out under license and in accordance with the ethical policies of the UK Home Office and the University of Cambridge, and in accordance with the relevant laws and regulations of the UK and EU. Experiments were approved by the University of Cambridge Animal Welfare Ethical Review Board (AWERB).

Cell Culture and Cellular Assays

Mouse splenic CD8$^+$ T-lymphocytes were isolated with anti-mouse CD8α microbeads (Miltenyi) on a MACS column. CD8$^+$ T-lymphocytes were activated with plate-bound αCD3 (5 μg/ml, Biolegend) and soluble αCD28 (1μg/ml, Biolegend) for 48 h. For activation of OT-I CD8$^+$ T-cells, total splenocytes from OT-I mice were cultured with SIINFEKL peptide (Sigma). Culture media was RPMI-1640 containing 2 mM glutamine (Gibco), 10% FBS, 25 mM HEPES, 1% penicillin-streptomycin (Gibco), 50 μM β-mercaptoethanol (Gibco). Following activation, CD8$^+$ T-lymphocytes were expanded for 5 days in the presence of 20 ng/ml recombinant murine IL-2 (Biolegend) and cultured under 21%, 5% or 1% oxygen conditions. For hypoxia experiments, cells were transferred into a Ruskinn Sci-tive hypoxia workstation.

LLC, MS-1 and B16-F10 cell lines were cultured at 37 C with high glucose DMEM (Gibco) supplemented with 10% FBS. B16-OVA culture media was supplemented with 0.8 mg/ml of G418 (Gibco).

Genomic Analyses of Breast Cancer Samples

Clinical cohorts: Gene-expression profiled breast cancer tissues from a population-based and well-annotated cohort of Swedish patients was used as previously described (the Uppsala cohort, n=251, (Miller et al., 2005). In brief, RNA was extracted from freshly frozen tumor tissue and profiled on Affymetrix HG-U133A and HG-U133B arrays. Affymetrix CEL files are available at the Gene Expression Omnibus Database under accession number GSE3494. Additionally, gene expression data from a translational substudy of a Phase 3 study of docetaxel with or without sunitinib as first line treatment of metastatic breast cancer was used (Bergh et al., 2012; Foukakis et al., 2015). In this study, transcriptomic profiling of metastatic tumor aspiration biopsies was carried out at baseline and 14 days after start of treatment (Foukakis et al., 2015), Gene Expression Omnibus Database accession number GSE54323. Last, in the Phase 2 PROMIX trial, patients (n=150) with locally advanced breast cancer were treated preoperatively with two cycles of chemotherapy with epirubicin and docetaxel, followed by four additional cycles of the same chemotherapy with the addition of bevacizumab (Clinicaltrials.gov identifier NCT00957125). Gene expression data at all three time points were available for 36 patients. The median age at the time of diagnosis was 49 years (range 33-65). The median tumor size at baseline was 56 mm (range 30-110). All tumors were HER2 negative, 24 of the tumors were ER positive and 11 ER negative (one with unknown ER status). After a median follow up time of 48 months, 8 patients had relapsed and 3 of them had died of breast cancer. Gene expression profiling of tumor core biopsies was performed at baseline, after two cycles of chemotherapy and at surgery (Gene Expression Omnibus Database accession number GSE87455). Informed consent was obtained from all subjects.

METHODS DETAILS

Metabolism Assays

Extracellular acidification rate (ECAR) and oxygen consumption rates (OCR) were measured using a XF24 3 Extracellular Flux Analyzer (Seahorse). 0.5 x 10^6 CD8$^+$ T cells per well were plated on poly-D-lysine coated plates, in XF media (non-buffered RPMI base medium, 25mM glucose, 2mM glutamine and 1 mM sodium pyruvate). Assays were performed with a Seahorse Cell Energy Phenotype Test Kit, and ECAR and OCR were measured simultaneously before and after injection of Olygomycin (1 μM) and FCCP (1 μM). For experiments performed under 1% O_2, the XF24 3 analyzer was placed inside a hypoxic globe box (Coy).

Glucose and lactate levels were determined with a Dade-Behring Dimension RXL autoanalyser (Siemens). VEGF-A levels in media were measured with a VEGF-A immunoassay kit (MSD). Protein levels were normalized to viable cell counts performed on an ADAM-MC automated cell counter (NanoEnTek). T cell proliferation was measured by CFSE dilution assay 72 hours after T cell activation with plate-bound αCD3 (5 μg/ml, Biolegend) and soluble αCD28 (1μg/ml, Biolegend).

Cytotoxicity Assays

The *in vitro* cytotoxicity assay shown in Figure S2I was performed by co-culturing EG7-OVA target cells with control or mutant OT-1 CD8$^+$ T cells, at different effector to target (E:T) ratios for 24 hours, under 21% or 1% O_2 . The percent cell specific killing of each CD8$^+$ T cell genotype was calculated by flow cytometry, and counting beads (ThermoFisher) were used as normalizing controls. The *in vitro*

antigen-specific killing assay shown in Figure S6E was performed using a 1:1 mix of CFSE labeled peptide-pulsed cells ($CFSE^{lo}$) and control cells ($CFSE^{hi}$) as targets. The percent cell specific killing of each $CD8^+$ T cell genotype was calculated based on the CFSE analysis by flow cytometry after a 4 hour co-culture of $CD8^+$ CTLs and target cells.

Transendothelial T-Cell Migration Assays

$25x10^4$ mutant or control α CD3/CD28 activated $CD8^+$ CTLs were subjected to 1% O_2 for 24 hours and then cultured in a Boyden chamber on top of a confluent layer of primary endothelial cells isolated from lungs of wild type mice, as described before (Branco-Price et al., 2012). 12 hours later T cells in the bottom well were counted.

Surface VCAM-1 and VE-Cadherin Analysis

MS-1 endothelial cells (ATCC) were cocultured with $VEGF^{fl/fl}dlck^{CRE}$ or $VEGF^{fl/fl}$ control CTLs for 2 hours, stained with fluorescent-labeled antibodies (Biolegend), and analyzed by flow cytometry. Recombinant mouse VEGF-A (Sigma) was used at 20 ng/mL.

Flow Cytometry

Cells were stained with LIVE/DEAD Violet (Life Technologies) before antibody staining. The following fluorophore-conjugated antibodies were used (Biolegend): α-CD62L, CD44, CD8, CD3, CD4, CD8, CD137, CD45.1, PD-1, OX40, GITR, TIM-3, LAG-3, Granzyme B, TNFα, IFNγ, CD25, and CD69. α-FOXP3 was from eBiosciences. For TNFα and IFNγ intracellular staining, cells were restimulated with SIINFEKL peptide (Sigma) for 4 hours in the presence of Golgi-stop (BD) or cell stimulation cocktail (eBiosciences) and then fixed and permeabilized with Fix/perm buffer (eBiosciences) before intracellular staining and acquisition on a Fortessa (BD). Data was analyzed by FlowJo (TreeStar).

For flow cytometric analyses of TILs, excised tumors were minced finely with a scalpel blade in a Petri dish, and incubated for 25 minutes at 37°C in Collagenase-D and DNAse-I (Roche) in RPMI before passage through a 70-μm cell strainer (BD) to obtain single-cell suspensions and perform antibody staining.

QRT-PCR and Western Blotting

Total RNA was extracted from isolated $CD8^+$ T cells (RNeasy kit, Qiagen) and 1 μg of RNA was used for cDNA synthesis (First-Strand Synthesis kit, Invitrogen). Tumor infiltrating macrophages and lymphocytes were positively isolated from single-cell suspensions with anti-mouse F4/80, CD8α or CD4 microbeads (Miltenyi) on a MACS column. Real time PCR was performed with SYBR green (Thermo) in a StepOnePlus system (Applied Biosystems). Samples were run in technical triplicates. Data was normalized to *18S rRNA* or *HPRT* expression. The primers used are shown in the Table S2.

To calculate the gDNA deletion efficiency, total DNA was extracted with the DNeasy kit (Qiagen) and the primers shown in the Table S3 were used in a Taqman RT-PCR mastermix (ThermoFisher).

For immunoblotting, nuclear and cytosolic fractions were prepared (NE-PER kit, Thermo Scientific), quantified by the DC-Protein Assay (BioRad), and separated by SDS–PAGE on a Nu-Page 3-8% Tris acetate gel (Life technologies). Proteins were transferred to PVDF trans-blot membranes (Bio-rad) and blocked in 5% milk prepared in phosphate-buffered saline (PBS) containing 0.05% Tween-20. Membranes were incubated with primary antibodies overnight at 4°C and horseradish peroxidase (HRP)-conjugated secondary antibody at RT, revealed with ECL Prime (GE Healthcare) and imaged with a Fusion FX analyser (Vilber Lourmat). The following primary antibodies were used at a dilution of 1:1000: HIF-1α (Novus), HIF-2α (Novus), HDAC1 (abcam), β-actin (abcam).

Tumor Growth

A total of 0.5×10^6 LLC or B16-F10 cells were injected subcutaneously into the flank in 100 μL of PBS. Mice and tumor size were monitored every other day and mice were sacrificed when tumor size reached 144 mm^2. Tumor sizes were measured using a digital caliper and tumor volumes calculated with the formula 1/2(length x $width^2$).

Adoptive Cell Transfer

Total splenocytes isolated from or OT-1-$VEGF^{fl/fl}dlck^{CRE}$ mice, and OT-1-HIF-$1\alpha^{fl/fl}$ or OT-1-$VEGF^{fl/fl}$ littermate controls were activated with SIINFEKL peptide in the presence of IL-2. Five days later, total splenocytes were inoculated intravenously ($1x10^6$ cells per mouse) into recipient mice carrying subcutaneous B16-OVA tumors ($5x10^5$ cells injected 5 days before T cell transfer). For the *in vivo* migration experiment, a 1:1 pool of mutant ($CD45.1^+TdTomato^+OT\text{-}1^+HIF\text{-}1\alpha^{fl/fl}dlck^{CRE}$) and control ($CD45.1^+OT\text{-}1^+HIF\text{-}1\alpha^{fl/fl}$) $CD8^+$ T cells ($1x10^6$ cells per genotype) was intravenously transferred into $CD45.2^+$ B16-OVA tumor-bearing WT mice. Spleens, lymph nodes (LN) and tumors were collected 48 hours after the co-transfer.

In Vivo Immunotherapy and Chemotherapy

Checkpoint blockade therapy consisting on a combination of 200 μg of αCTLA-4 (clone 9H10, BioXCell) and 200 μg of αPD-1 (RMP1-14, BioXCell) antibodies per dose or isotype control antibodies (Syrian hamster IgG and Rat IgG2a, respectively, BioXCell) was administered on days 5, 7 and 9 after tumor injection, which consisted on $0.5x10^6$ MC38 colon carcinoma cells injected subcutaneously.

The treatment with chemotherapy was administered on days 5, 7 and 9 after the subcutaneous injection of $0.5x10^6$ LLC tumor cells. Cyclophosphamide (i.p., 170mg/kg) was purchased from Sigma.

Histology, Immunofluorescence and *In Vivo* pO_2 Measurements

Mammary glands were collected and fixed in 4% paraformaldehyde (Santa Cruz Biotechnology) for 24 hours, washed in 70% ethanol and embedded in paraffin. 10 μm sections were deparaffinized with Histoclear II (National diagnostics) and rehydrated in graded ethanol. Sections were stained with haematoxylin/eosin and scored histologically as previously described (Lin et al., 2003). For vascular staining, each mouse was injected intravenously with 100 μg of DyLight 594 labeled Lycopersicon Esculentum lectin (Vector laboratories) 5 minutes before sacrifice. 10 μm sections were mounted with ProLong Gold antifade mountant with DAPI (ThermoFisher) and photographed with a Leica DMRB microscope. Vessel analyses were performed with ImageJ and a customized software application from Wimasis. For pimonidazole staining, mice were injected with 60 mg/kg bodyweight of pimonidazole hydrochloride (hpi Hypoxyprobe), 1 hour before tumor collection. Tissues were embedded in paraffin and pimonidazole adducts were detected with a FITC-conjugated mouse IgG1 antibody at a 1:50 dilution (hpi Hypoxyprobe). Mouse α-SMA antibody (Sigma) was used at a 1:100 dilution followed by anti-mouse AF-488 secondary antibody (Life technologies). Complete tumor section pictures were taken by tiling acquisition with a Leica TCS SPE confocal microscope and hypoxic area percentages and pericyte coverage were calculated with ImageJ.

Dissolved oxygen (pO_2) was measured in complete cell culture medium under 21%, 5% and 1% O_2 in a Ruskinn cell culture workstation, and in the spleens and LLC subcutaneous tumors implanted in WT mice (n=2), by an Oxylite sensor equipped with a needle-encased sensor. Two-tailed student T test was used for comparisons.

STATISTICAL ANALYSIS

Statistical Analysis

Data were analyzed in GraphPad Prism 6 software. Pairwise comparisons were performed using an unpaired Student's t-test. Grouped data were assessed by two-way ANOVA with Bonferroni's correction, to adjust for multiple comparisons. Tumor volumes were analyzed by two-way ANOVA followed by Sidak correction for multiple comparisons. Survival curves were compared with the long-rank (Mantel-Cox) test. Statistical details of experiments can be found in the figure legends. n represents number of animals. p values legend is: ****<0.00005, ***< 0.0005, **<0.005, *< 0.05, n.s.=not significant.

Clinical Gene-Expression Data Analysis

All analysis of the clinical gene-expression data was performed in R/Bioconductor. Probe intensities were extracted from Affymetrix CEL files and background corrected, log2-transformed, normalized and summarized to probeset expression using the rma function in the affy package with the default settings. Probesets were mapped to Entrez Gene ID and symbol using the hgu133a.db and hgu133b.db annotation packages. When multiple probesets were mapped to the same Entrez Gene ID, the average expression was used. PAM50 intrinsic subtype classification was performed. Statistical analysis: Survival analysis of the clinical gene-expression data was performed in R using the survival package with default settings.

Cancer Cell, Volume *32*

Supplemental Information

An HIF-1α/VEGF-A Axis in Cytotoxic T Cells Regulates Tumor Progression

Asis Palazon, Petros A. Tyrakis, David Macias, Pedro Veliça, Helene Rundqvist, Susan Fitzpatrick, Nikola Vojnovic, Anthony T. Phan, Niklas Loman, Ingrid Hedenfalk, Thomas Hatschek, John Lövrot, Theodoros Foukakis, Ananda W. Goldrath, Jonas Bergh, and Randall S. Johnson

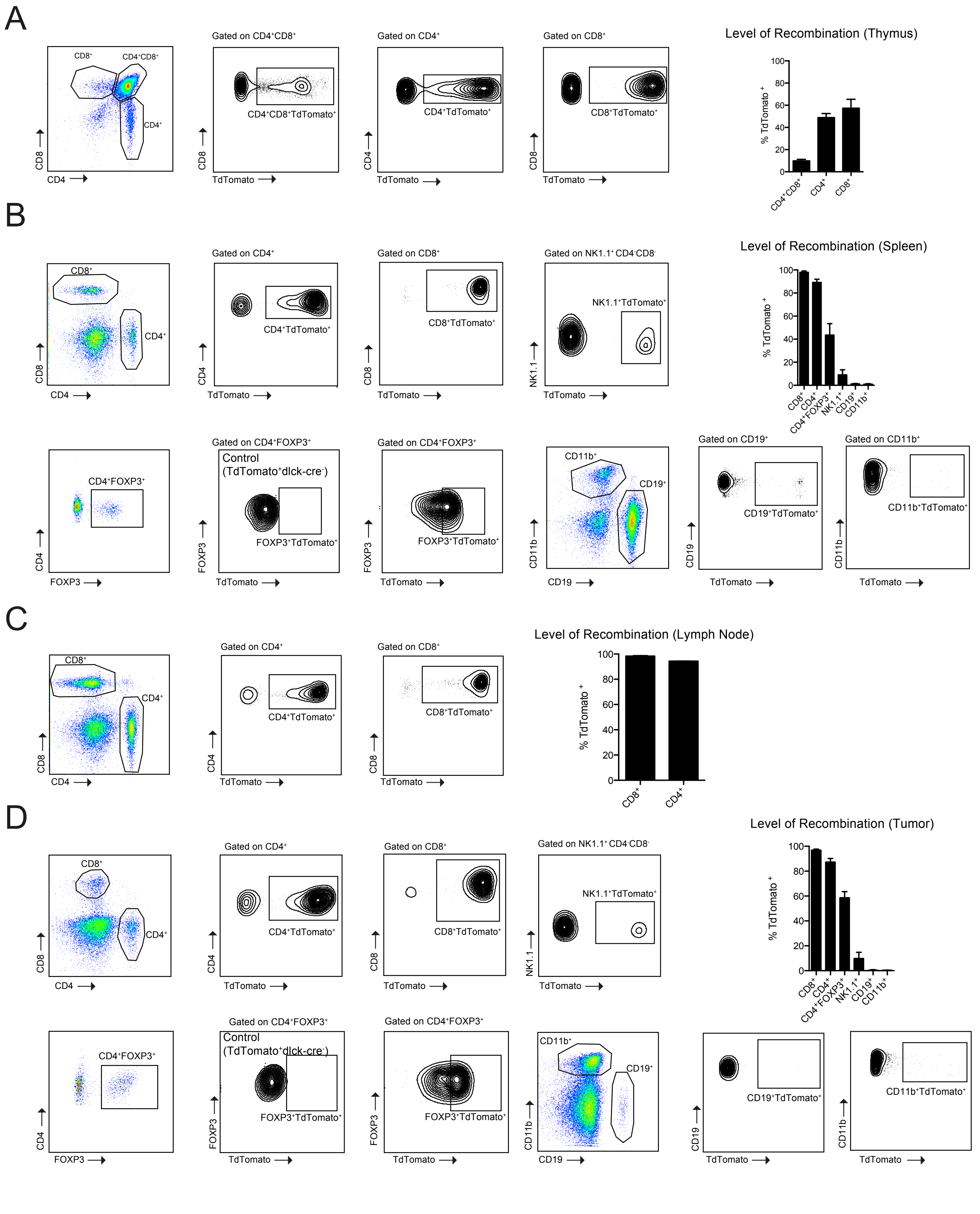

Figure S1, related to Figure 1.

(A-D) Level of dlck-cre recombination in thymus (A), spleen (B), lymph nodes (C), and subcutaneous LLC tumors (D) (n=4) was measured using a TdTomato reporter mouse line by flow cytometry. Error bars represent SD.

Table S1, related to Figure 1.

	Mean pO_2 (mmHg)	SD
Culture media 21% O_2	107.38	2.65
Culture media 5% O_2	31.65	0.26
Culture media 1% O_2	10.33	4.25
Spleen	21.73	12.6
Tumor (LLC)	1.95	2.41

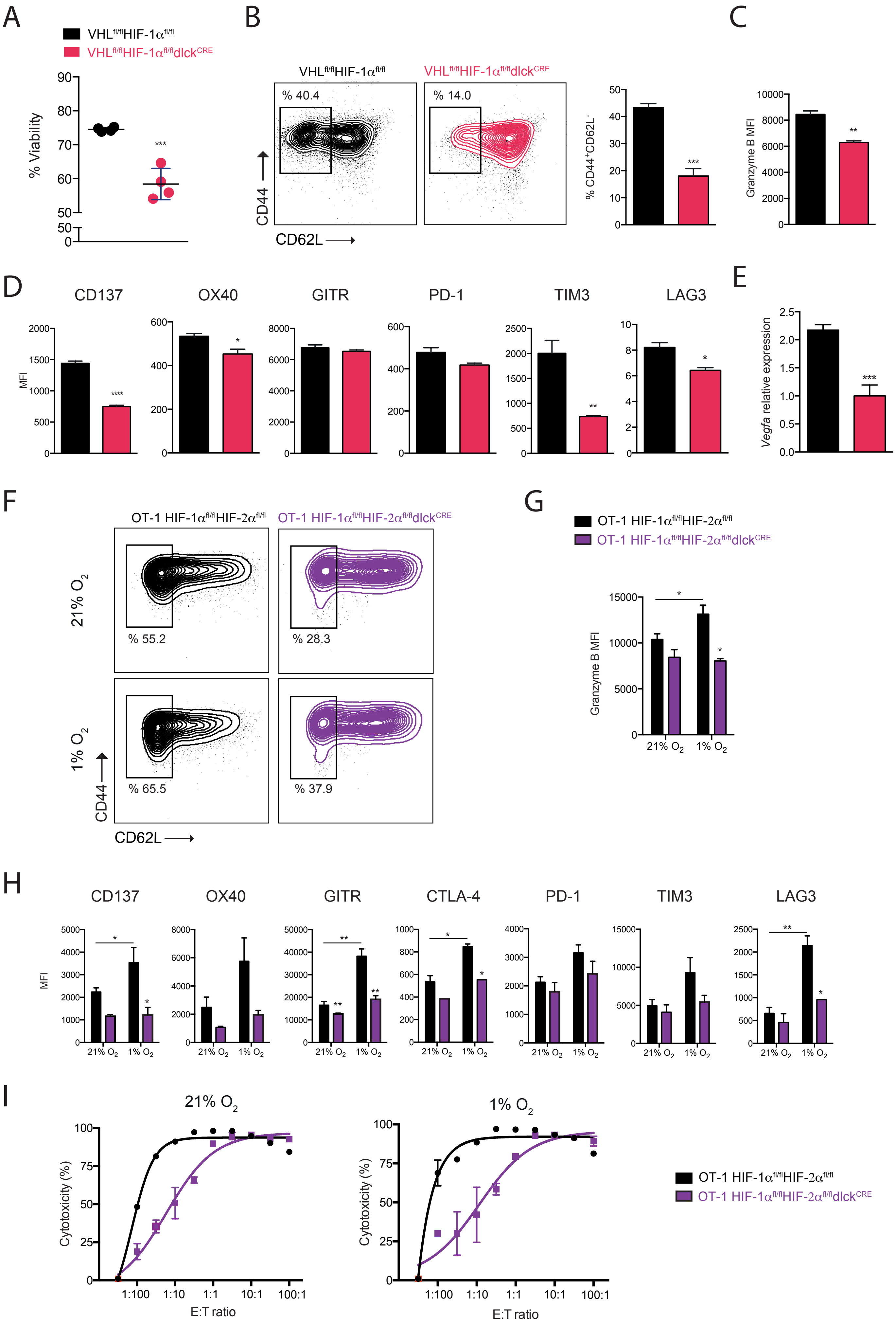

A
VHLfl/flHIF-1αfl/fl
VHLfl/flHIF-1αfl/fldlckCRE
% Viability
***
B
VHLfl/flHIF-1αfl/fl
VHLfl/flHIF-1αfl/fldlckCRE
% 40.4
% 14.0
CD44
CD62L
% CD44+CD62L-
***
C
Granzyme B MFI
**
D
CD137
OX40
GITR
PD-1
TIM3
LAG3
MFI
****
*
**
*
E
Vegfa relative expression
***
F
OT-1 HIF-1αfl/flHIF-2αfl/fl
OT-1 HIF-1αfl/flHIF-2αfl/fldlckCRE
21% O2
1% O2
% 55.2
% 28.3
% 65.5
% 37.9
CD44
CD62L
G
OT-1 HIF-1αfl/flHIF-2αfl/fl
OT-1 HIF-1αfl/flHIF-2αfl/fldlckCRE
Granzyme B MFI
21% O2
1% O2
H
CD137
OX40
GITR
CTLA-4
PD-1
TIM3
LAG3
MFI
21% O2
1% O2
I
21% O2
1% O2
Cytotoxicity (%)
E:T ratio
1:100
1:10
1:1
10:1
100:1
OT-1 HIF-1αfl/flHIF-2αfl/fl
OT-1 HIF-1αfl/flHIF-2αfl/fldlckCRE

Figure S2, related to Figure 2.
(A-E) $CD8^+$ T cells were isolated from spleens of $VHL^{fl/fl}HIF\text{-}1\alpha^{fl/fl}dlck^{CRE}$ (pink) and littermate control (black) mice, activated with αCD3/αCD28 for 48 hr, and then expanded for 5 days in the presence of IL-2: percent survival (A), expression of CD44 and CD62L by flow cytometry (B), expression of Granzyme B by intracellular flow cytometry (C), surface expression of costimulatory molecules/checkpoint receptors CD137, OX40, GITR, PD-1, TIM-3 and LAG3 by flow cytometry (D) and relative expression of *Vegfa* by QRT-PCR (E), n=4, error bars represent SD. (F-H) $CD8^+$ T cells were isolated from spleens of OT-1 $HIF\text{-}1\alpha^{fl/fl}HIF\text{-}2\alpha^{fl/fl}dlck^{CRE}$ (purple) and littermate control (black) mice, activated with cognate peptide for 2 days, and then expanded for 5 days in the presence of IL-2 and subjected to 21% or 1% O_2 for 24 hr: expression of CD44 and CD62L by flow cytometry (F) expression of Granzyme B by intracellular flow cytometry (G), expression of the indicated costimulatory molecules/checkpoint receptors by flow cytometry (H), n=4, error bars represent SD. (I) *In vitro* cytotoxicity assay: EG7-OVA target cells and control or mutant OT-1 $CD8^+$ T cells were co-cultured at different effector to target (E:T) ratios for 24 hr under 21% or 1% O_2 (n=2, error bars represent SE).

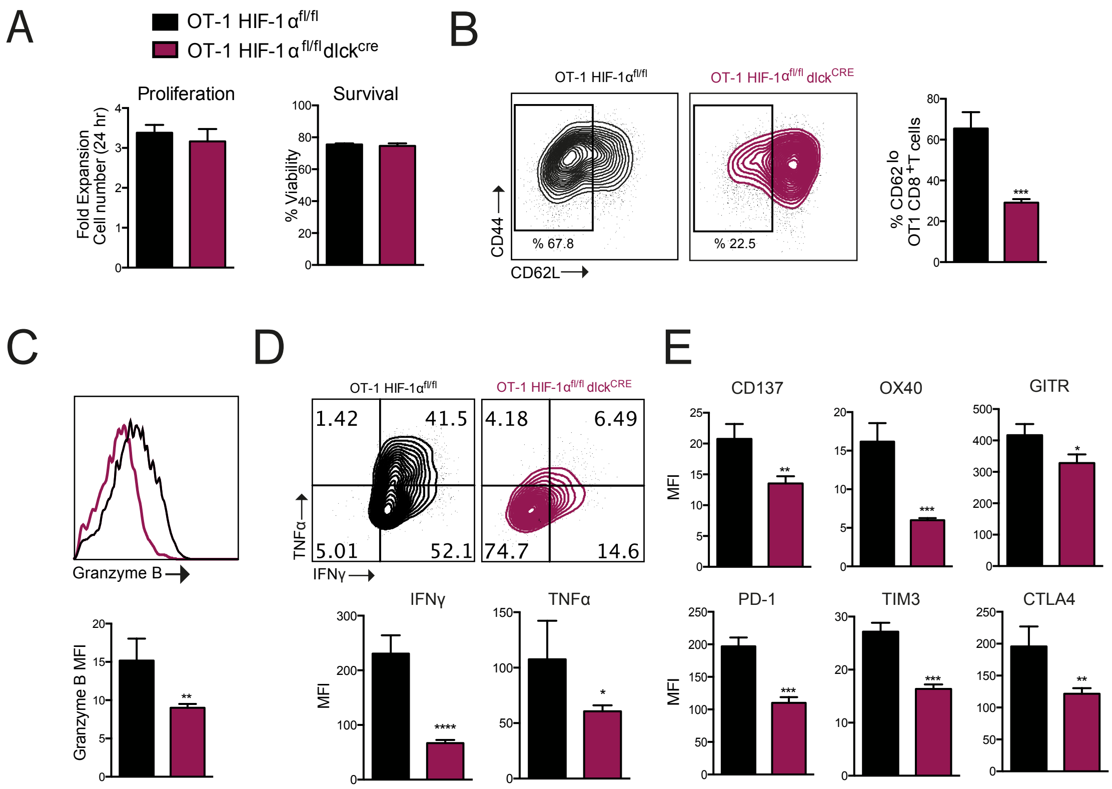

A
OT-1 HIF-1α fl/fl
OT-1 HIF-1α fl/fl dlckcre
Proliferation
Fold Expansion Cell number (24 hr)
Survival
% Viability
B
OT-1 HIF-1αfl/fl
OT-1 HIF-1αfl/fl dlckCRE
CD44
CD62L
% 67.8
% 22.5
% CD62 lo OT1 CD8+T cells
C
Granzyme B
Granzyme B MFI
D
OT-1 HIF-1αfl/fl
OT-1 HIF-1αfl/fl dlckCRE
1.42
41.5
5.01
52.1
4.18
6.49
74.7
14.6
TNFα
IFNγ
MFI
E
CD137
OX40
GITR
PD-1
TIM3
CTLA4
MFI

Figure S3, related to Figure 4.

(A-B) Splenic OT-1 cells were activated *in vitro* with cognate SIINFEKL peptide and expanded under 21% O_2 for 3 days in the presence of IL-2: proliferation and survival (A), surface CD62L expression by flow cytometry (B), (n=3, error bars represent SD). (C) Intracellular granzyme B expression (n=3, error bars represent SD). (D) Intracellular TNFα and IFNγ expression after 4 hr *in vitro* restimulation with SIINFEKL (n=3, error bars represent SD). (E) Surface expression of costimulatory/checkpoint receptors CD137, OX40, GITR, PD-1 and TIM3 and intracellular expression of CTLA4 (n=3, error bars represent SD). Two-tailed student T test was used for comparisons.

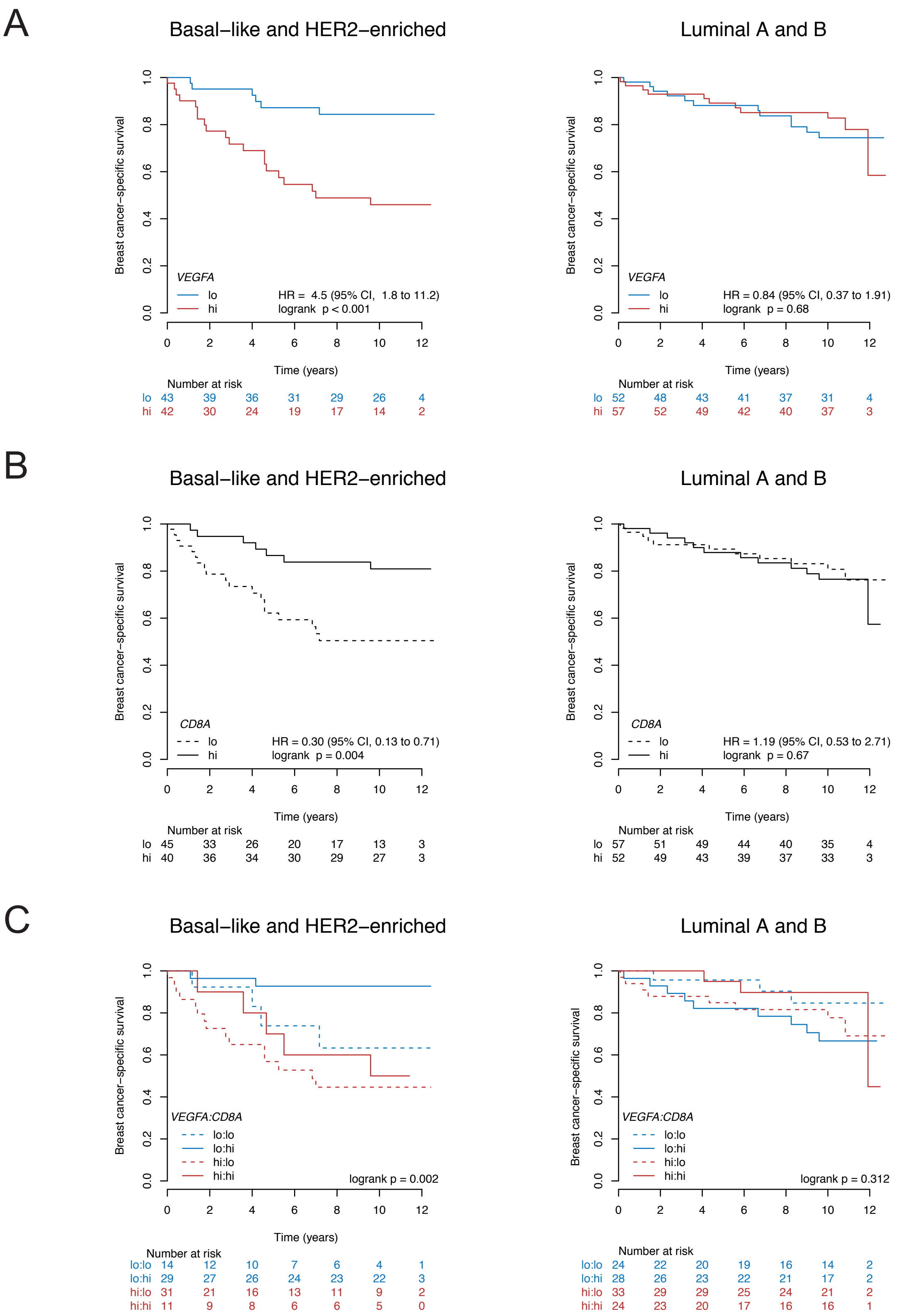
A
Basal–like and HER2–enriched
Breast cancer–specific survival
VEGFA
lo
hi
HR = 4.5 (95% CI, 1.8 to 11.2)
logrank p < 0.001
Time (years)
Number at risk
lo 43 39 36 31 29 26 4
hi 42 30 24 19 17 14 2
Luminal A and B
Breast cancer–specific survival
VEGFA
lo
hi
HR = 0.84 (95% CI, 0.37 to 1.91)
logrank p = 0.68
Time (years)
Number at risk
lo 52 48 43 41 37 31 4
hi 57 52 49 42 40 37 3
B
Basal–like and HER2–enriched
Breast cancer–specific survival
CD8A
lo
hi
HR = 0.30 (95% CI, 0.13 to 0.71)
logrank p = 0.004
Time (years)
Number at risk
lo 45 33 26 20 17 13 3
hi 40 36 34 30 29 27 3
Luminal A and B
Breast cancer–specific survival
CD8A
lo
hi
HR = 1.19 (95% CI, 0.53 to 2.71)
logrank p = 0.67
Time (years)
Number at risk
lo 57 51 49 44 40 35 4
hi 52 49 43 39 37 33 3
C
Basal–like and HER2–enriched
Breast cancer–specific survival
VEGFA:CD8A
lo:lo
lo:hi
hi:lo
hi:hi
logrank p = 0.002
Time (years)
Number at risk
lo:lo 14 12 10 7 6 4 1
lo:hi 29 27 26 24 23 22 3
hi:lo 31 21 16 13 11 9 2
hi:hi 11 9 8 6 6 5 0
Luminal A and B
Breast cancer–specific survival
VEGFA:CD8A
lo:lo
lo:hi
hi:lo
hi:hi
logrank p = 0.312
Number at risk
lo:lo 24 22 20 19 16 14 2
lo:hi 28 26 23 22 21 17 2
hi:lo 33 29 29 25 24 21 2
hi:hi 24 23 20 17 16 16 1

Figure S4, related to Figure 5.
(A-C) Association of *VEGFA* and *CD8A* expression with clinical outcome in the Uppsala cohort. Kaplan-Meyer survival curves of breast-cancer specific survival are shown for the indicated subtypes, for patients classified according to *VEGFA* relative expression levels (A), *CD8A* relative expression levels (B), and different ratios of *VEGFA*:*CD8A* relative expression levels (C).

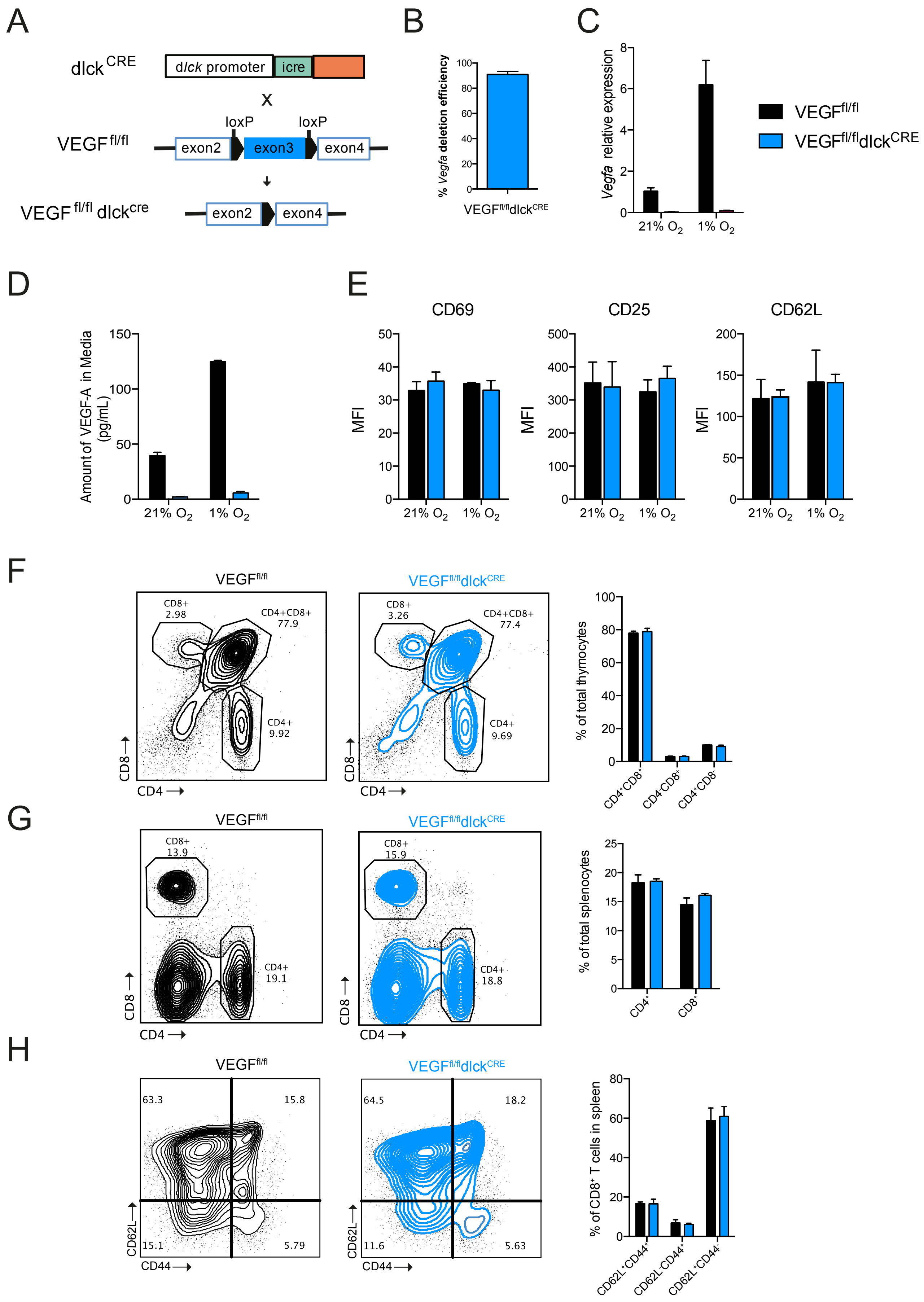
A
dlck CRE
dlck promoter
icre
x
loxP
loxP
VEGF fl/fl
exon2
exon3
exon4
VEGF fl/fl dlckcre
exon2
exon4
B
% Vegfa deletion efficiency
VEGFfl/fldlckCRE
C
Vegfa relative expression
21% O2
1% O2
VEGFfl/fl
VEGFfl/fldlckCRE
D
Amount of VEGF-A in Media (pg/mL)
21% O2
1% O2
E
CD69
CD25
CD62L
MFI
21% O2
1% O2
F
VEGFfl/fl
VEGFfl/fldlckCRE
CD8+
2.98
CD4+CD8+
77.9
CD4+
9.92
CD8+
3.26
CD4+CD8+
77.4
CD4+
9.69
CD8
CD4
% of total thymocytes
CD4+CD8+
CD4-CD8+
CD4+CD8-
G
VEGFfl/fl
VEGFfl/fldlckCRE
CD8+
13.9
CD4+
19.1
CD8+
15.9
CD4+
18.8
% of total splenocytes
CD4+
CD8+
H
VEGFfl/fl
VEGFfl/fldlckCRE
63.3
15.8
15.1
5.79
64.5
18.2
11.6
5.63
CD62L
CD44
% of CD8+ T cells in spleen
CD62L+CD44+
CD62L-CD44+
CD62L+CD44-

Figure S5, related to Figure 6.

(A) Schematic diagram and gene deletion strategy to generate $VEGF^{fl/fl}dlck^{CRE}$ mice. (B) Percentage of *Vegfa* deletion efficiency in gDNA from $CD8^{+}$ CTLs generated *in vitro* after isolation from from $VEGF^{fl/fl}dlck^{CRE}$ mice (n=4, error bars represent SD). (C) Relative expression of *Vegfa* by QRT-PCR on CTLs from mutant and control mice subjected to 24 hr of culture under 21% O_2 or 1% O_2 (n=4, error bars represent SD). (D) Amount of VEGF-A in media of $CD8^{+}$ CTLs cultured as in (C), (n=4, error bars represent SD). (E) Mean fluorescence intensity for the surface expression of CD69, CD25 and CD62L measured by flow cytometry on $CD8^{+}$ T cells 48 hr after activation with αCD3/αCD28. (n=4, error bars represent SD). (F) Thymic composition of mutant mice ($VEGF^{fl/fl}dlck^{CRE}$) and control littermates ($VEGF^{fl/fl}$)(n=4, error bars represent SD). (G) Splenic composition of mutant mice ($VEGF^{fl/fl}dlck^{CRE}$) and control littermates ($VEGF^{fl/fl}$) (n=4, error bars represent SD). (H) $CD8^{+}$ T cell composition in spleen of mutant mice ($VEGF^{fl/fl}dlck^{CRE}$) and control littermates ($VEGF^{fl/fl}$) according to CD62L and CD44 expression(n=4, error bars represent SD).

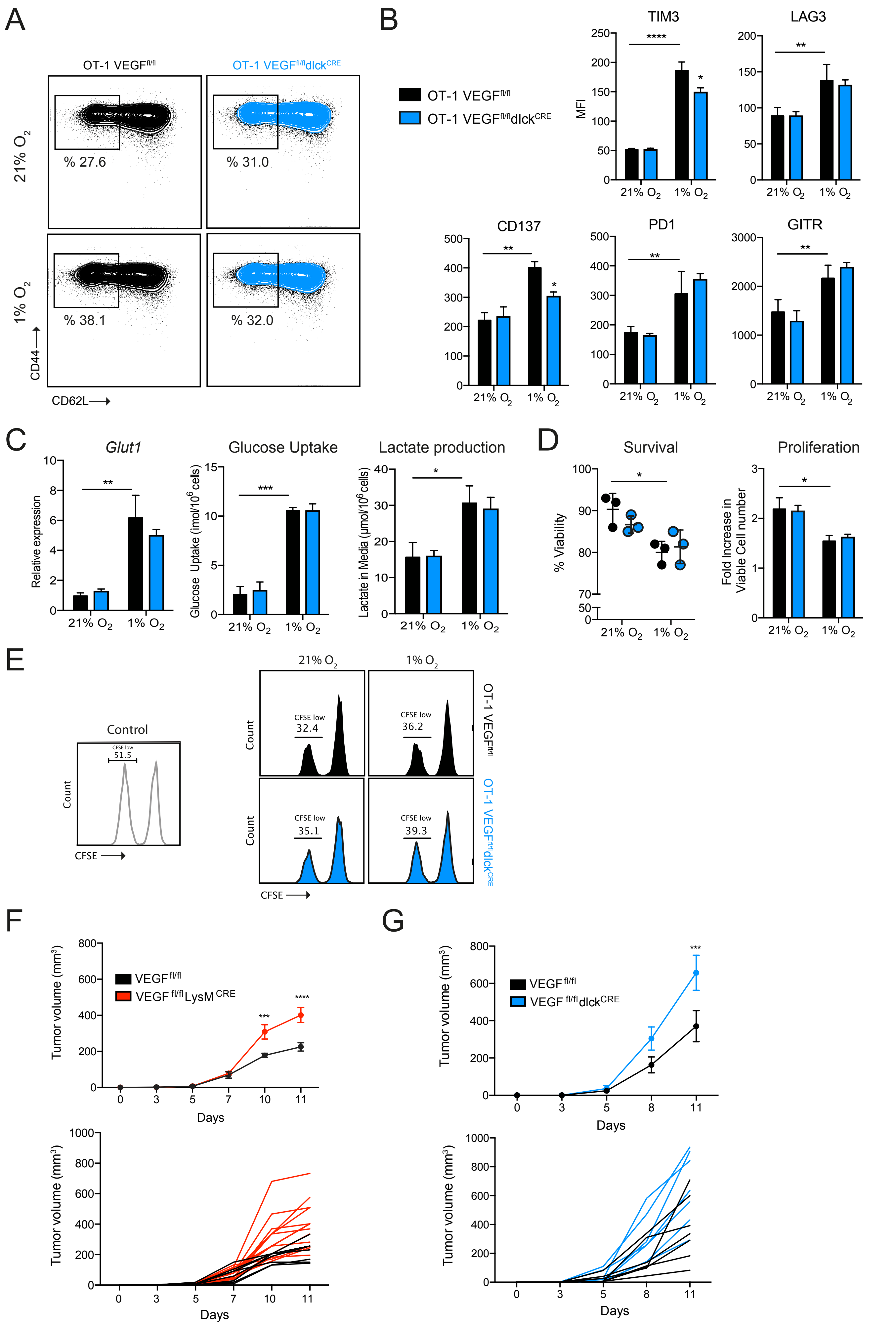

A
OT-1 VEGFfl/fl
OT-1 VEGFfl/fldlckCRE
21% O2
1% O2
% 27.6
% 31.0
% 38.1
% 32.0
CD44
CD62L
B
OT-1 VEGFfl/fl
OT-1 VEGFfl/fldlckCRE
TIM3
LAG3
CD137
PD1
GITR
MFI
21% O2
1% O2
C
Glut1
Glucose Uptake
Lactate production
Relative expression
Glucose Uptake (μmol/10^6 cells)
Lactate in Media (μmol/10^6 cells)
D
Survival
Proliferation
% Viability
Fold Increase in Viable Cell number
E
Control
CFSE low
51.5
21% O2
1% O2
32.4
36.2
35.1
39.3
Count
CFSE
OT-1 VEGFfl/fl
OT-1 VEGFfl/fldlckCRE
F
VEGFfl/fl
VEGF fl/flLysM CRE
Tumor volume (mm3)
Days
G
VEGFfl/fl
VEGF fl/fldlckCRE
Tumor volume (mm3)
Days

H

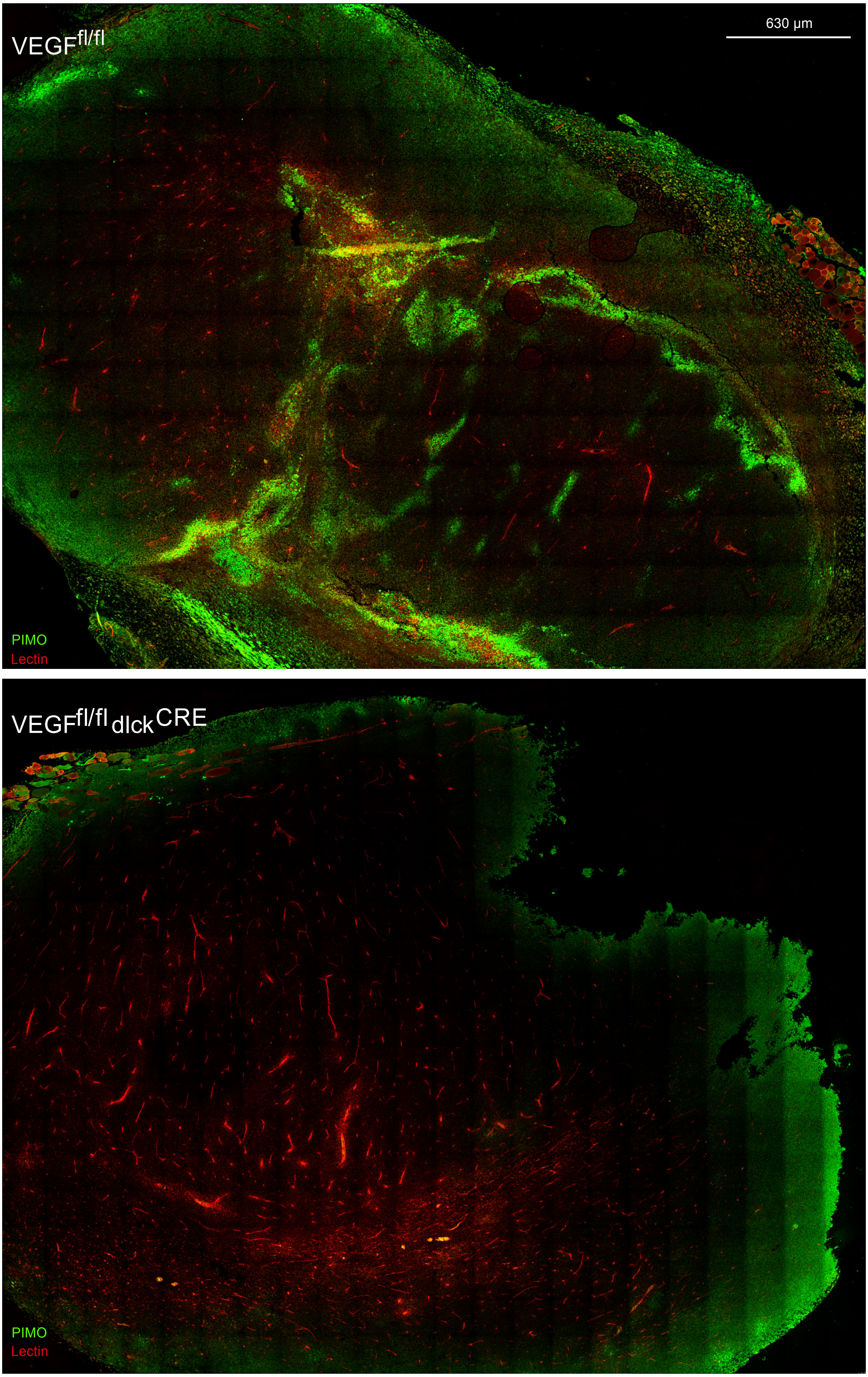

Figure S6, related to Figure 6

(A-D) Splenic $CD8^+$ T cells were isolated from mutant mice (OT-1 $VEGF^{fl/fl}dlck^{CRE}$) and control littermates (OT-1 $VEGF^{fl/fl}$), activated *in vitro* with cognate SIINFEKL peptide and expanded in the presence of IL-2 under 21% O_2 for 5 days and subjected to 21% O_2 or 1% O_2 for the last 24 hours: surface CD62L and CD44 expression by flow cytometry (A), surface expression of costimulatory/checkpoint receptors TIM3, LAG3, CD137, PD-1 and GITR (B), relative mRNA expression of Glut-1 on CTLs (C, Left), glucose uptake and lactate production by CTLs with the indicated genotypes (C, Right) and percent survival (D, Left) and proliferation (D, Right). (n=4, error bars represent SD). (E) *In vitro* killing assay: target cells were pulsed with peptide and stained with $CFSE^{lo}$ and mixed in equal proportions with unpulsed $CFSE^{hi}$ cells. CTLs from mutant or control mice were added to the culture for 4 hours and % of $CFSE^+$ cells were quantified by flow cytometry. (F-G) Representative growth curves of subcutaneously injected LLC tumors in $VEGF^{fl/fl}LysM^{CRE}$ (red, n=13) (F) and $VEGF^{fl/fl}dlck^{CRE}$ (blue, n=7) (G). $VEGF^{fl/fl}$ littermate controls are shown in black, ($0.5x10^6$ tumor cells/mouse). Error bars represent SEM, statistical analysis was performed by two-way ANOVA with Sidak correction for multiple comparisons. (H) Representative immunofluorescence images from LLC tumors for the indicated genotypes (Green= pimonidazole, Red= Tomato lectin-Dylight-594).

Table S2, related to STAR Methods. Primers for qRT-PCR.

Gene	Forward primer	Reverse primer
Rn18s	5'-CGGCGACGACCCATTCGAAC-3'	5'-GAATCGAACCCTGATTCCCCGTC-3'
Hprt	5'- TCAGTCAACGGGGGACATAAA-3'	5'- GGGGCTGTACTGCTTAACCAG-3'
Pgk1	5'-ATTCTGCTTGGACAATGGAGC-3'	5'-AGGCATGGGAACACCATCA-3'
Hk2	5′-TGATCGCCTGCTTATTCACGG-3′	5'-AACCGCCTAGAAATCTCCAGA-3'
Pdk1	5'-GAAGCAGTTCCTGGACTTCG-3'	5'-CCAACTTTGCACCAGCTGTA-3'
Vegfa	5'-CCACGTCAGAGAGCAACATCA-3'	5'-TCATTCTCTCTATGTGCTGGCTTT-3'
Mct4	5'-TCACGGGTTTCTCCTACGC-3'	5'-GCCAAAGCGGTTCACACAC-3'
Hif1a	5'-GAAACGACCACTGCTAAGGCA-3'	5'-GGCAGACAGCTTAAGGCTCCT-3'
Epas1	5'-CAACCTGCAGCCTCAGTGT-3'	5'-CACCACGTCGTTCTTCTCGA-3'

Table S3, related to STAR Methods. Primers for determining deletion efficiency.

Actb Forward	5'-AGAGGGAAATCGTGCGTGA-3'
Actb Reverse	5'-CAATAGTGATGACCTGGCCGT-3'
Actb Probe	5'-[6-FAM] CACTGCCGCATCCTCTTCCTC [BHQ1a-Q]-3'
Vegfa Forward	5'-TGACCATCTGCTTTCGTGACC-3'
Vegfa Reverse	5'-ACTTGTTGCAGGCAGCGG-3'
Vegfa Probe	5'-[6-FAM] TGCTCCCTGGGCTCGACAGGG [BHQ1a-Q]-3'
Hif1a Forward	5' GGTGCTGGTGTCCAAAATGTAG 3'
Hif1a Reverse	5' ATGGGTCTAGAGAGATAGCTCCACA 3'
Hif1a Probe	5' [6-FAM] CCTGTTGGTTGCGCAGCAAGCATT [BHQ1a-Q] 3'
Hif2a Forward	5' TCTATGAGTTGGCTCATGAGTTG 3'
Hif2a Reverse	5' GTCCGAAGGAAGCTGATGG 3'
Hif2a Probe	5' [6-FAM] CCACCTGGA/ZEN/CAAAGCCTCCATCAT [3IABkFQ] 3'
